# Brain Tumor Classification in MRI Images Using Combined Transfer Learning and Convolutional Neural Networks

**DOI:** 10.3390/jimaging12060233

**Published:** 2026-05-28

**Authors:** Maisam Abbas, Muhammad Hassan, Ran-Zan Wang, Chin-Hung Teng

**Affiliations:** 1 Department of Computer Science and Engineering, Yuan Ze University, Yuandong Rd., Zhongli District, Taoyuan 32003, Taiwan; s1129105@mail.yzu.edu.tw (M.A.); s1126062@mail.yzu.edu.tw (M.H.); 2 Department of Information Communication, Yuan Ze University, Yuandong Rd., Zhongli District, Taoyuan 32003, Taiwan; chteng@saturn.yzu.edu.tw

**Keywords:** brain tumor classification, Magnetic Resonance Imaging (MRI), convolutional neural network, transfer learning, pre-trained models

## Abstract

Early and accurate brain tumor detection is vital for effective treatment. We propose a deep learning framework for MRI-based brain tumor classification, featuring a novel Custom CNN evaluated independently alongside six pre-trained models for comparative analysis (InceptionV3, EfficientNetV2L, ResNet152V2, Xception, VGG16, and MobileNetV2). Additionally, three separate ensemble models are constructed to analyze whether model combination improves performance. Experiments conducted on the Kaggle-Multiclass brain MRI dataset show that the proposed Custom CNN achieves the best performance, with an accuracy of 99.54%, and features a task-specific architecture (0.57M parameters) that achieves superior performance through domain-specific feature learning and computational efficiency, thus outperforming both individual pre-trained models and ensemble approaches. Among pre-trained models, EfficientNetV2L (99.47%) and InceptionV3 (99.39%) show competitive results, while the best ensemble model achieves 99.47% accuracy, indicating clinical deployment potential pending external validation. These results demonstrate that the proposed Custom CNN provides superior performance without requiring ensemble complexity, thus highlighting its effectiveness and efficiency for automated brain tumor classification.

## 1. Introduction

Brain tumors represent critical pathological conditions characterized by abnormal cell proliferation within brain tissues, posing significant challenges to global healthcare systems [1]. The World Health Organization classifies over 120 tumor types spanning from benign (Grade I) to highly malignant (Grade IV) categories [2]. Among these, gliomas, meningiomas, and pituitary tumors are predominant, with gliomas being the most aggressive (45% of cases) and meningiomas the most prevalent benign tumors (53.9% of non-malignant cases) [3].

Early and accurate detection is paramount for effective treatment planning and improved patient outcomes [4]. Traditional diagnosis relies on manual MRI interpretation by radiologists, a process that is time-consuming, subjective, and prone to errors, especially with large datasets or subtle tumor characteristics [5]. MRI has emerged as the preferred imaging modality due to its non-invasive nature, superior soft tissue contrast, and absence of ionizing radiation [6]. However, the complexity of MRI scans and intricate tumor grading systems necessitate specialized expertise, often causing diagnostic delays [7].

Artificial intelligence and deep learning have revolutionized medical image analysis, offering enhanced diagnostic accuracy and efficiency [8]. Convolutional Neural Networks (CNNs) excel in automatically learning hierarchical features from raw images, making them highly effective for classification, segmentation, and feature extraction tasks [9]. Unlike traditional machine learning requiring hand-crafted features, deep learning models automatically identify both general and specialized characteristics in medical images [10].

Transfer learning has proven particularly valuable in medical imaging; it enables fine-tuning of pre-trained models for specific diagnostic tasks [11]. This approach reduces computational costs and training time while achieving high performance, which is especially beneficial given limited medical datasets [12]. State-of-the-art architectures, including VGG-16, ResNet-50, InceptionV3, DenseNet121, EfficientNet variants, and Xception, have been successfully adapted for brain tumor classification [13]. Recent research has increasingly focused on ensemble learning approaches that combine multiple models to leverage complementary strengths and improve performance [14].Multi-path and multi-scale CNN architectures have also gained attention for capturing diverse spatial and contextual features through varying kernel sizes [15].

Despite these advances, significant challenges persist in current methodologies, including limited generalizability across datasets, inadequate performance in resource-constrained environments, and vulnerability to noisy or imbalanced data [16]. The complexity of brain tumor classification demands sophisticated architectures capable of distinguishing subtle morphological differences while maintaining computational efficiency [17]. Advanced preprocessing techniques, including CLAHE, gamma correction, and multi-view analysis, have shown promise in addressing some limitations [18], yet comprehensive comparative studies evaluating Custom CNNs against multiple pre-trained models and ensemble approaches remain lacking.

However, most existing ensemble and transfer learning pipelines prioritize marginal accuracy gains at the expense of computational overhead, limiting their feasibility for real-time clinical deployment. Furthermore, the absence of standardized interpretability mechanisms hinders radiologist trust, quality assurance, and regulatory approval. A systematic evaluation that jointly optimizes diagnostic performance, parameter efficiency, and decision transparency remains unaddressed in the current literature.

To address these limitations, we propose a comprehensive approach combining a meticulously crafted Custom CNN with six state-of-the-art pre-trained architectures (InceptionV3, EfficientNetV2L, ResNet152V2, Xception, VGG16, and MobileNetV2) and three strategically developed ensemble models. Our approach leverages the synergistic combination of ensemble learning and transfer learning techniques to maximize classification accuracy while maintaining computational efficiency for clinical deployment. Unlike conventional ensemble strategies that aggregate heavy architectures, our framework explicitly balances accuracy with resource constraints, employing probabilistic fusion only when complementary feature representations justify the computational trade-off. Model interpretability is further enforced through gradient-based activation mapping to validate anatomical grounding and facilitate clinical auditability.

The main contributions of this research include: (1) developing an optimized Custom CNN architecture specifically designed for brain tumor classification, achieving state-of-the-art performance with 99.54% accuracy; (2) evaluating and comparing six pre-trained deep learning models and three ensemble strategies on a standardized Kaggle-Multiclass brain MRI dataset; (3) demonstrating that task-specific CNN design can outperform generic transfer learning models and ensemble approaches; (4) establishing new performance benchmarks for automated brain tumor diagnosis, with our Custom CNN significantly outperforming existing state-of-the-art methods; (5) introducing a reproducible, tri-criteria evaluation framework (accuracy–efficiency–interpretability) that enables evidence-based model selection for clinical deployment.

## 2. Related Work

The field of brain tumor classification from MRI has evolved from traditional machine learning to sophisticated deep learning methodologies, with recent emphasis on ensemble transfer learning techniques for enhanced diagnostic accuracy.

### 2.1. Traditional Machine Learning Approaches and Their Limitations

Early investigations in automated brain tumor detection predominantly relied on conventional machine learning techniques with manual feature extraction. Faragallah et al. [19] pioneered Otsu binarization with K-means clustering for tumor segmentation, employing DWT and PCA for feature extraction and dimensionality reduction, utilizing SVM for classification. Similarly, Ahmed et al. [20] implemented GLCM and DWT-based feature extraction with probabilistic neural networks, achieving approximately 100% accuracy in distinguishing normal and abnormal tissues. These traditional methodologies were inherently constrained by their dependence on hand-crafted feature engineering, proving to be both time-consuming and susceptible to human error [21]. The reliance on manually designed features necessitated accurate tumor localization and exhibited high susceptibility to mistakes, thus highlighting the need for more robust automated approaches [22].

### 2.2. Deep Learning Revolution in Medical Imaging

The advent of CNNs fundamentally transformed medical image analysis, particularly in brain tumor classification. Abiwinanda et al. [23] evaluated five distinct CNN architectures, with their second architecture achieving 98.51% training accuracy and 84.19% validation accuracy. Pashaei et al. [24] introduced KE-CNN, combining CNNs with KELM for multi-class classification, achieving 93.68% accuracy. Huang et al. [25] proposed CNN-BCN, incorporating modified activation functions and random graph generation algorithms, achieving 0.5–0.75% improvement in test accuracy. Sultan et al. [26] developed a sixteen-layered CNN achieving 96.13% test accuracy on multi-class classification and 98.7% on glioma grading tasks. Zulfiqar et al. [27] extended this work with multi-pathway CNNs for simultaneous classification and segmentation, attaining 97.3% test accuracy.

### 2.3. Transfer Learning Paradigms and Pre-Trained Model Utilization

Transfer learning has emerged as pivotal in addressing limited medical imaging datasets and computational constraints. Noreen et al. [28] pioneered pre-trained Inception-V3 and DenseNet201 architectures with feature concatenation strategies, achieving 99.34% and 99.51% accuracies, respectively. Swati et al. [29] evaluated AlexNet, VGG16, and VGG19, with VGG19 achieving 94.82% test accuracy. Shaikh et al. [7] implemented a pre-trained GoogLeNet with Softmax, SVM, and K-NN classifiers. Rajput et al. [30] applied transfer learning with pre-trained CNNs, achieving 90% average accuracy. Sajjad et al. [31] utilized deep CNNs with data augmentation, attaining 94.5% accuracy, while Maharjan et al. [32] enhanced performance with modified SoftMax loss functions. Asif et al. [33] employed multiple architectures including Xception, DenseNet201, DenseNet121, ResNet152V2, and InceptionResNetV2 with custom softmax configurations. Hossain et al. [34] demonstrated ensemble approaches combining VGG16, InceptionV3, VGG19, ResNet50, InceptionResNetV2, and Xception, achieving peak accuracies ranging from 93.58% to 96.94%.

### 2.4. Ensemble Learning Methodologies

Ensemble learning represents a significant advancement, addressing single-model limitations through complementary architecture integration. Minarno et al. [35] implemented CNN models with hyperparameter tuning, achieving 96.00% accuracy on Kaggle datasets, while Noreen et al. [28] applied ensemble methods on Figshare datasets, attaining 94.34% accuracy. Singh et al. [36] utilized ensemble methods on Figshare datasets, achieving 96.08% accuracy. Shah et al. [37] applied deep CNN models on BR35H datasets, demonstrating 97.86% accuracy for binary classification, while Islam et al. [38] implemented CNN-LSTM hybrid architectures, achieving 97.93% accuracy. Khan et al. [13] proposed Hybrid-NET models on Figshare datasets, achieving 93.36% accuracy, highlighting the ongoing balance between accuracy improvements and computational overhead. Devnath et al. [39] further validated the utility of probabilistic fusion strategies, simple averaging, weighted averaging, and majority voting, for medical image classification, demonstrating that ensemble consensus can enhance robustness without proportional increases in architectural complexity.

### 2.5. Advanced Architectures for Brain Tumor Classification

Recent advancements have focused on hybrid and optimized architectures. Alzahrani [40] developed ConvAttenMixer, integrating convolutional layers, self-attention, and external attention mechanisms, achieving 97.94% accuracy. Meshram et al. [41] explored Swin Transformers, demonstrating transformer architecture potential in medical imaging. Bhuvaneswari Ramakrishnan et al. [42] proposed optimized hybrid CNN architectures using OneAPI techniques. Rastogi et al. [43] developed multi-branch networks with inception blocks, implementing five-fold cross-validation. Hekmat et al. [44] introduced differential evolution-driven optimized ensemble networks, showing improved performance through metaheuristic optimization. These studies highlight a shift toward sophisticated models combining CNNs, transformers, and advanced optimization techniques.

### 2.6. Research Gaps and Contemporary Challenges

Despite significant progress, critical limitations remain. Many studies utilize outdated or small datasets, hindering model generalizability [45]. The lack of ensemble approaches limits performance gains beyond individual architectures [46]. Computational efficiency remains challenging, with promising techniques often too slow for clinical use [47]. CNNs like VGG16 and ResNet-50 show reduced robustness without ensemble methods [48]. Models combining SVMs with CNNs demonstrate varied performance but are constrained by limited datasets, leading to potential overfitting [49]. Despite high reported metrics for CNN-LSTM architectures, generalizability concerns persist due to small datasets [50]. These gaps highlight the need for robust, efficient ensemble methods combining transfer learning and CNNs to improve accuracy and generalizability in brain tumor classification.

This work integrates transfer learning, ensembling, and interpretability into a unified, reproducible framework under clinical deployment constraints. It jointly optimizes diagnostic accuracy, computational efficiency, and decision transparency for resource-constrained settings. The standardized benchmarking protocol and open-source implementation enhance reproducibility and enable fair comparison in medical AI.

## 3. Materials and Methods

This section outlines the methodology for brain tumor classification in MRI images using combined transfer learning and Custom CNNs. The process begins with data preprocessing, including resizing and normalization of MRI images to ensure consistent input for model training.

We employed six pre-trained CNN architectures (InceptionV3, EfficientNetV2L, ResNet152V2, Xception, VGG16, and MobileNetV2) utilizing transfer learning to adapt them for our specific classification task. These models were fine-tuned to address class imbalance and specialize in distinguishing tumor types. A Custom CNN was developed featuring four convolutional layers with increasing filter sizes (64, 64, 128, 128), each followed by max pooling. The extracted features were processed through two dense layers before a softmax output layer for multi-class classification. The network was optimized using the Adam optimizer with specific beta parameters ($\beta_{1}=0.85$, $\beta_{2}=0.9925$) to enhance convergence and stability.

All models were trained using a uniform dataset split and identical epoch length for comparative evaluation. Performance metrics, including accuracy, loss curves, and training time, were recorded. This comparative approach was used to identify the most effective individual architecture, combining the strengths of transfer learning and purpose-built CNNs for robust brain tumor classification.

### 3.1. Dataset Description and Characteristics

The dataset employed in this study is a carefully compiled collection of human brain MRI scans, sourced from three reputable repositories: Figshare [51], SARTAJ dataset [52], and BR35H [53]. This comprehensive dataset comprises 7023 MRI images from the Kaggle-Multiclass dataset [54], systematically organized into four clinically significant categories: Glioma, Meningioma, Pituitary, and No Tumor. The training subset includes 1321, 1339, 1457, and 1595 images for each respective class, while the testing set consists of 300, 306, 300, and 405 images, as detailed in Table 1. Table 2 summarizes the performance of various pre-trained CNN models, highlighting their comparative classification accuracy on the Kaggle-Multiclass dataset.

**Figure 1:**
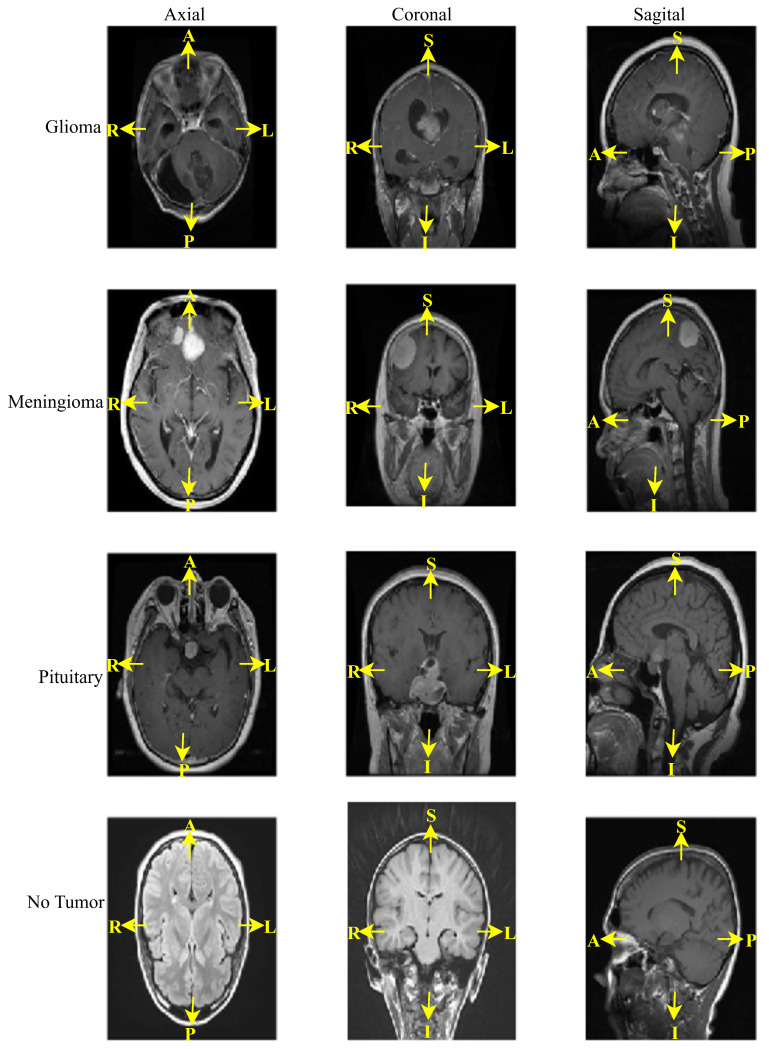
Representative MRI scans from the Kaggle multiclass brain tumor dataset across axial, coronal, and sagittal views. Yellow arrows indicate anatomical orientation directions—A (anterior), P (posterior), R (right), L (left), S (superior), and I (inferior)—ensuring spatial interpretability of brain regions.

**Figure 2:**
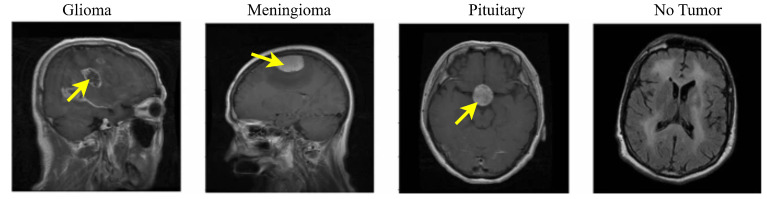
Class-wise representative MRI samples from the dataset showing Glioma, Meningioma, Pituitary, and No Tumor cases across different anatomical planes. Yellow arrows indicate the presence and location of tumor regions within each MRI class, highlighting the affected areas for improved visual interpretation and class-wise distinction.

This dataset encompasses four classes: Gliomas (malignant glial cell tumors), Meningiomas (typically benign meningeal tumors), Pituitary tumors (benign or malignant pituitary gland masses), and No Tumor (normal brain images). Its diverse representation of tumor morphologies, spatial distributions, and intensity profiles enhances model generalizability and robustness. Figure 1 and Figure 2 illustrate the visual heterogeneity of the Kaggle-Multiclass dataset across axial, coronal, and sagittal views, annotated with standard anatomical orientation markers (A: anterior, P: posterior, R: right, L: left, S: superior, I: inferior), with arrows indicating the tumor regions.

### 3.2. Comprehensive Data Preprocessing for Pre-Trained and Custom CNN Architectures

To ensure optimal performance across multiple CNN architectures, the MRI dataset underwent a systematic preprocessing pipeline. The following steps were executed:

#### 3.2.1. Image Resizing

All MRI scans were resized to 224×224 pixels using bilinear interpolation with zero-padding to preserve anatomical proportions where possible. While this standardization enables compatibility with ImageNet pre-trained architectures, we acknowledge that aspect-ratio-preserving resizing may further improve geometric fidelity and will be prioritized in future work [55]:

(1) $$I_{\mathrm{resized}}\left(x,y\right)=\sum_{i,j}I_{\mathrm{original}}\left(i,j\right)\cdot w\left(i,j,x,y\right)$$

#### 3.2.2. Normalization

Pixel intensities were scaled to the range [0,1] using min–max normalization:

(2) $$I_{\mathrm{norm}}\left(x,y\right)=\frac{I_{\mathrm{original}}\left(x,y\right)-I_{\min}}{I_{\max}-I_{\min}}$$

For 8-bit grayscale images, this simplifies to:

(3) $$I_{\mathrm{norm}}\left(x,y\right)=\frac{I_{\mathrm{original}}\left(x,y\right)}{255}$$

#### 3.2.3. Data Shuffling

Training samples were randomly shuffled using a bijective mapping function to eliminate bias due to data ordering:

(4) $$D_{\mathrm{shuffled}}={\left\{\left(X_{\sigma (i)},y_{\sigma (i)}\right)\right\}}_{i=1}^{N}$$

where σ is a random permutation function.

#### 3.2.4. Data Augmentation

To enhance model generalization and implicitly regularize against MRI noise, real-time spatial and photometric transformations were applied:

- Horizontal Flip: Applied along the sagittal plane:
  (5) $$I_{\mathrm{flipped}}\left(x,y\right)=I\left(W-x,y\right)$$
- Vertical Flip: Applied along the axial plane:
  (6) $$I_{\mathrm{flipped}}\left(x,y\right)=I\left(x,H-y\right)$$
- Rotation: Angular variation with $\theta ∼U(-20^{\circ },20^{\circ })$:
  (7) $$\left[\begin{array}{c} x^{\prime }\\ y^{\prime } \end{array}\right]=\left[\begin{array}{cc} \cos\theta & -\sin\theta \\ \sin\theta & \cos\theta \end{array}\right]\left[\begin{array}{c} x\\ y \end{array}\right]$$
- Zooming: Scaling factor s∼U(0.8,1.2):
  (8) $$I_{\mathrm{zoom}}\left(x,y\right)=I\left(\frac{x}{s}+\delta_{x},\frac{y}{s}+\delta_{y}\right)$$
- Contrast Adjustment: Contrast factor c∼U(0.8,1.2):
  (9) $$I_{\mathrm{contrast}}\left(x,y\right)=\mathrm{clip}\left(c\cdot I_{\mathrm{norm}}\left(x,y\right),0,1\right)$$
- Translation: Offsets $\Delta_{x},\Delta_{y}∼U\left(-22,22\right)$:
  (10) $$I_{\mathrm{translated}}\left(x,y\right)=I\left(x-\Delta_{x},y-\Delta_{y}\right)$$

While min–max normalization provides basic intensity standardization, the extensive augmentation pipeline described above serves as an implicit regularizer against MRI-specific noise artifacts (e.g., Rician noise, bias field inhomogeneity) by exposing the model to diverse perturbations during training. This approach reduces overfitting to scanner-specific noise patterns without requiring explicit denoising pre-processing [56].

The resulting training loss incorporates an implicit regularization term:

(11) $$L_{\mathrm{aug}}=E_{(x,y)∼D}\left[E_{t∼T}\left[\ell \left(f_{\theta }\left(t\left(x\right)\right),y\right)\right]\right]$$

**Figure 3:**
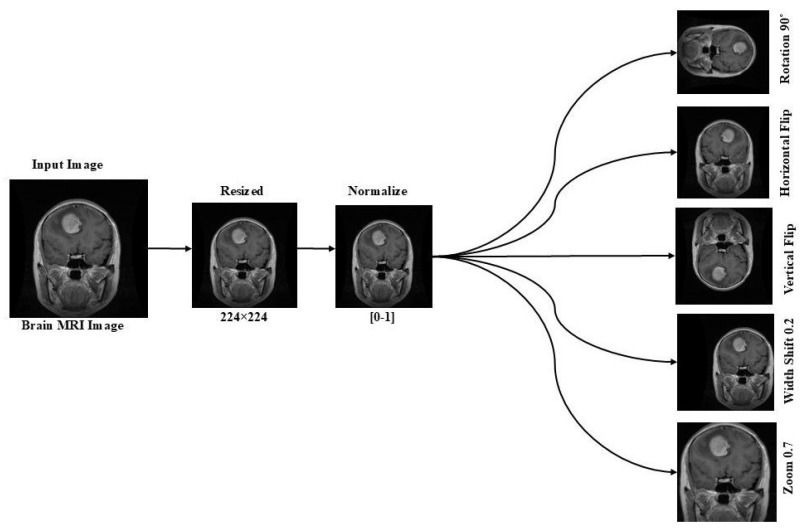
Overview of the MRI preprocessing and data augmentation pipeline, including input image, resizing, normalization, intensity enhancement, and geometric transformations to improve model generalization.

The complete preprocessing pipeline, illustrated in Figure 3, was uniformly applied across all experiments to ensure consistent data quality and fair model comparison.

#### 3.2.5. Addressing Data Limitations

Despite the relatively large size of the dataset, potential limitations, such as data imbalance, variability in acquisition conditions, and limited clinical diversity, were carefully addressed in this study. First, the dataset integrates MRI scans from multiple publicly available sources, introducing heterogeneity in imaging characteristics and improving generalization capability. Second, extensive data augmentation techniques were applied, including rotation, translation, scaling, flipping, and contrast adjustments, effectively increasing the diversity of training samples and reducing the risk of overfitting. Third, transfer learning was utilized through multiple pre-trained models, enabling the extraction of robust and generalized feature representations learned from large-scale datasets. Finally, consistent training protocols, uniform data splits, and identical evaluation metrics were used across all models to ensure fair comparison and reduce experimental bias. These strategies collectively mitigate the impact of data limitations and enhance the reliability of the proposed approach.

### 3.3. Customized CNN and Base Models for Feature Extraction

To effectively classify brain tumors in MRI images, we designed a compact custom convolutional neural network (CNN) and leveraged transfer learning from established architectures. Given the limited size of the Kaggle-Multiclass brain tumor dataset, transfer learning provides a more efficient and effective approach by utilizing pre-trained models to extract high-level semantic features.

**Figure 4:**
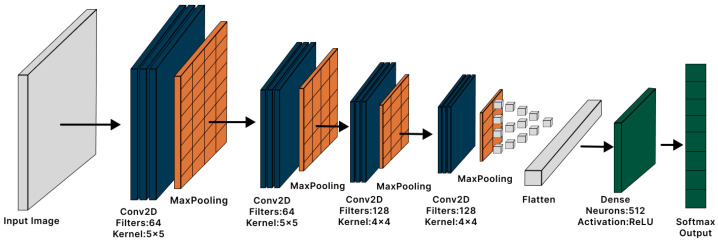
The proposed custom convolutional neural network (CNN) architecture, illustrating feature extraction blocks, convolutional layers, pooling operations, and fully connected classification layers.

The Custom CNN architecture, as illustrated in Figure 4, consists of four convolutional layers with increasing filter sizes (64, 64, 128, 128) and decreasing kernel sizes (5×5, 5×5, 4×4, 4×4), each followed by max-pooling layers to reduce spatial dimensions. The extracted features are flattened and passed through a fully connected layer with 512 ReLU-activated neurons before reaching the softmax output layer for multi-class classification. The model contains 0.57 million trainable parameters, spans 11 layers, and occupies only 6.52 MB of disk space. It is trained using the Adam optimizer with a learning rate of 0.001 and beta values of 0.85 and 0.9925. In parallel, several well-known pre-trained CNN models were fine-tuned and evaluated systematically to identify the best-performing base models for integration into our ensemble framework. These models serve as powerful feature extractors, significantly improving classification accuracy while reducing training time and computational cost compared to training from scratch.

The Custom CNN was intentionally developed as a lightweight, task-specific baseline to contextualize the computational efficiency of the proposed ensemble. While the primary objective targets ensemble-driven diagnostic accuracy, this architecture serves as a necessary reference point to quantify the accuracy-versus-resource trade-off. Its inclusion enables (1) direct benchmarking of domain-tailored feature extraction against multi-model transfer learning and (2) isolation of the marginal performance gains attributable to ensemble complexity versus architectural simplicity. This aligns with our efficiency-focused objective by ensuring model selection is grounded in empirical cost-benefit analysis rather than architectural scale alone.

### 3.4. Graphical Abstract of the Proposed Transfer Learning-Based Ensemble Framework

**Figure 5:**
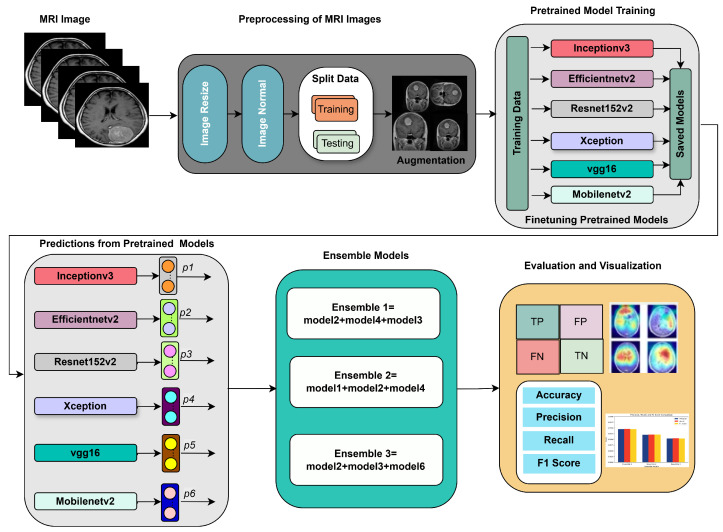
Overview of the proposed transfer learning-based ensemble framework, including MRI preprocessing, feature extraction using multiple pretrained models, fine-tuning, ensemble learning, and final classification.

Figure 5 presents the graphical abstract of the proposed framework, where MRI images are used as input and subjected to preprocessing, including resizing, normalization, and augmentation. The preprocessed data are then utilized to fine-tune multiple pre-trained models for extracting discriminative features, followed by a selective ensembling strategy that combines complementary models. The final classification performance is evaluated using standard metrics, including accuracy, precision, recall, and F1-score.

#### 3.4.1. InceptionV3 Architecture

InceptionV3 [57] is a powerful convolutional neural network architecture widely used in image recognition tasks. Unlike sequential models, InceptionV3 employs a parallel multi-branch architecture that extracts multiscale features simultaneously through convolutional branches with different kernel sizes (1 × 1, 3 × 3, 5 × 5) and pooling operations.

The architecture incorporates factorization strategies, decomposing 5 × 5 convolutions into two consecutive 3 × 3 convolutions to reduce computational complexity while maintaining representational power. Auxiliary classifiers at intermediate layers help stabilize training by mitigating vanishing gradients. Batch normalization, ReLU activations, and average pooling enhance convergence and generalization.

InceptionV3 balances depth, width, and computational efficiency, achieving high accuracy with approximately 21.83 million parameters and a model size of 262 MB. In our study, InceptionV3 serves as Model 1, providing robust diagnostic performance for brain MRI abnormality classification while maintaining operational feasibility.

#### 3.4.2. EfficientNetV2-L Architecture

EfficientNetV2-L [58] is a state-of-the-art convolutional neural network architecture designed for large-scale image recognition tasks. The architecture employs inverted residual bottleneck blocks and squeeze-and-excitation mechanisms to learn rich, multiscale representations while maintaining computational efficiency.

During training, adaptive class weights were applied to address dataset imbalance: 1.0482 for class 0, 1.0964 for class 1, 0.89678 for class 2, and 0.98153 for class 3. This approach ensures balanced learning across all diagnostic categories and prevents overfitting to dominant classes. Batch normalization and ReLU activations further enhance convergence and generalization.

EfficientNetV2-L balances depth, width, and computational efficiency, achieving a high accuracy-to-parameter ratio suitable for complex medical image recognition. In our study, EfficientNetV2-L serves as Model 2 with approximately 118.4 million parameters (117.87 million trainable, 0.53 million non-trainable) and a model size of 1323.04 MB. Training spanned 50 epochs over 10,643.72 s, achieving 99.47% accuracy with 0.0291 testing loss, demonstrating robust performance for automated brain MRI abnormality detection.

#### 3.4.3. ResNet152V2 Architecture

ResNet152V2 builds upon He et al.’s [59] residual learning framework, featuring a 152-layer deep network with 59.4 million parameters and a 680 MB disk footprint. The residual skip connections enable effective gradient flow across deep layers, maintaining structural integrity during brain tumor MRI analysis.

Despite its depth, the architecture demonstrated efficient training performance over 50 epochs (1.73 h), achieving 99.89% training accuracy and 98.25% validation performance. Class weighting (coefficients: 0.897–1.096) enhanced discrimination across tumor categories, resulting in 98.47% test accuracy with 0.059 loss. The terminal learning rate of $1\times 10^{-6}$ ensured stable convergence while capturing subtle morphological features critical for brain tumor classification.

The model’s memory investment is justified by its diagnostic-grade performance, which effectively preserves tumor boundary delineation and tissue heterogeneity even in challenging MRI slices. This capability stems from the residual mapping innovations that maintain spatial hierarchies across deep layers, making it particularly suitable for complex neuroanatomical pattern recognition in brain tumor diagnosis.

#### 3.4.4. Xception Architecture

The Xception architecture leverages Szegedy et al.’s [60] depthwise separable convolutions, offering remarkable efficiency in brain tumor MRI classification with a streamlined 250 MB framework. The model utilizes 21.9 million total parameters (83.59 MB), including 21.8 million trainable parameters (99.62%) fine-tuned for MRI classification and 83,040 non-trainable parameters (0.38%) from base configurations.

The architecture’s extreme inception modules decouple spatial and cross-channel correlations, enabling efficient feature extraction with minimal computational overhead. Training completed in 1.22 h (85 s/epoch), achieving 99.9% training precision and 97.81% validation precision. Class rebalancing with weights of 1.096 for underrepresented categories enhanced performance.

Final testing yielded 98.17% accuracy with 0.065 loss, demonstrating robust generalizability despite having 27% fewer parameters than ResNet152V2. Terminal convergence at $1\times 10^{-6}$ learning rate prevented overfitting while capturing subtle morphological features critical for brain tumor classification. The model’s lightweight design maintains forensic precision in low-information regions, making it suitable for resource-constrained environments requiring rapid, reliable diagnosis.

#### 3.4.5. VGG16 Architecture

The VGG16 architecture exhibits parametric efficiency with 14.98 million total parameters (57.14 MB memory footprint), despite its 162 MB disk presence due to training metadata. The configuration includes 13.24 million trainable parameters (88.4%) actively refining brain tumor MRI features and 1.74 million non-trainable parameters (11.6%) preserving ImageNet transfer learning benefits.

During 1.26-h training, VGG16 achieved 99.86% training accuracy using Simonyan and Zisserman’s [61] sequential 3 × 3 convolutional stacks. Class weighting (1.096 for minority classes) addressed dataset imbalances, though validation loss (0.1563) indicated limitations in handling MRI artifacts. Final testing yielded 97.33% accuracy with 0.142 loss, the highest among benchmarked models.

VGG16’s 561ms inference latency, 23% slower than Xception despite fewer parameters, demonstrates classical architecture inefficiencies. Each million parameters delivers 0.65% accuracy versus Xception’s 0.86%, reflecting modern advantages in depthwise separable convolutions. Nonetheless, its 97.33% accuracy remains clinically viable for deployment-constrained environments where state-of-the-art models are impractical.

#### 3.4.6. MobileNetV2 Architecture

MobileNetV2 [62] demonstrates exceptional parametric efficiency in neuroimaging analysis with only 2.92 million total parameters (11.12 MB), an order of magnitude leaner than conventional models while maintaining diagnostic accuracy. The framework allocates 2.88 million trainable parameters (10.98 MB) for pathological brain pattern extraction and 37,472 non-trainable parameters (146.38 KB) for structural stability, resulting in a 33.7 MB trained model.

During 1.02-h training (50 epochs at 510 ms/step), the model achieved 99.87% training accuracy and 97.81% validation accuracy. Class reweighting (coefficients: 0.897–1.096) addressed dataset imbalances, yielding 97.94% test accuracy with 0.084 loss. Inverted residual blocks with linear bottleneck layers enabled robust performance while achieving 139 ms inference latency per scan, 3 to 4 times faster than heavier models.

MobileNetV2’s 11.12 MB profile represents an optimal balance for edge-deployed medical imaging, delivering VGG16-equivalent accuracy with 84% fewer parameters. Depthwise separable convolutions reduce computational redundancy while preserving hierarchical feature learning in T1/T2-weighted sequences. The 97.94% diagnostic precision, validated against various MRI artifacts and orientations, confirms that lightweight architectures can match heavyweight counterparts in clinical brain image evaluation when optimally engineered.

### 3.5. Models Computational Complexity Analysis

Table 3 presents a comparative analysis of seven CNN architectures evaluated across key computational metrics: trainable parameters, memory consumption, training time, and network depth.

EfficientNetV2-L is the most computationally intensive, with 118.4 million parameters across 480 layers, requiring 1323.04 MB memory. Its compound scaling and squeeze-and-excitation mechanisms provide exceptional representational capacity for high-precision diagnostics. ResNet152V2 follows with 59.4 million parameters across 152 layers and a 680 MB memory footprint, utilizing residual learning for stable deep training.

InceptionV3 maintains a balanced profile with 21.83 million parameters over 313 layers and 262 MB memory, employing multi-branch architectures and filter factorization for optimized depth and computational load. Xception features 21.91 million parameters and 126 layers with depthwise separable convolutions, achieving efficient feature extraction in 250 MB memory.

VGG16 employs a straightforward 3 × 3 convolutional approach with 14.98 million parameters across 41 layers, consuming 162 MB memory but lacking modern architectural innovations. MobileNetV2 excels in efficiency with 2.92 million parameters and 157 layers in just 33.7 MB, using inverted residuals and linear bottlenecks for resource-constrained deployment.

The proposed Custom CNN demonstrates the most compact architecture with 0.57 million parameters, 11 layers, and 6.52 MB memory consumption. It completes training in 473.01 s, highlighting its potential for real-time clinical deployment where computational constraints are stringent yet reliable diagnostic performance is essential.

### 3.6. Design and Implementation of the Proposed Ensemble Model

The proposed framework employs a multi-stream ensemble strategy to enhance brain MRI classification accuracy by leveraging the complementary strengths of diverse CNN architectures. Initially, a pool of five high-performing pre-trained models, InceptionV3, EfficientNetV2L, ResNet152V2, Xception, and MobileNetV2, was considered based on their strong performance in visual recognition tasks. From this pool, three distinct ensemble configurations were constructed, where each ensemble consists of three heterogeneous models. This design ensures a balance between predictive diversity, computational efficiency, and model robustness.

Recognizing individual model limitations in capturing comprehensive spatial and contextual features, three distinct ensemble configurations were constructed using high-performing pre-trained models: InceptionV3, EfficientNetV2, ResNet152V2, Xception, and MobileNetV2, each fine-tuned on curated brain MRI data as shown in Figure 5.

These pre-trained models were selected for their architectural diversity and proven efficacy in visual recognition tasks. EfficientNetV2’s compound scaling, InceptionV3’s multi-path convolutions, and ResNet152V2’s deep residual learning provide orthogonal advantages in extracting anatomical and pathological cues from MRI scans. Each ensemble combines three heterogeneous networks to capitalize on synergies between shallow/deep features, wide/narrow receptive fields, and residual/dense connectivity paradigms.

The ensemble mechanism operates at the decision level using probabilistic fusion through averaging of softmax probability distributions from constituent models. For each input MRI scan, the final prediction reflects model consensus, mathematically defined as:

(12) $$P_{\mathrm{ensemble}}\left(C\right)=\frac{1}{n}\sum_{i=1}^{n}P_{i}\left(C\right)$$

where n=3 is the number of models in each ensemble and $P_{i}\left(C\right)$ denotes the softmax probability for class *C* from the v*i*th model.

Notably, not all candidate models were included in every ensemble configuration. In particular, EfficientNetV2L, despite its strong performance, was selectively excluded from certain ensembles due to its significantly higher computational cost and training time, as reported in Table 3. Including such heavy architectures in all ensembles would increase redundancy and reduce efficiency without proportional performance gain.

The three ensemble configurations were systematically designed to evaluate performance across distinct computational tiers while maintaining a consistent high-performing anchor model (Model-2) across all setups. This anchor ensures stable baseline predictions, enabling controlled isolation of how architectural diversity impacts consensus stability under varying resource constraints. This ablation-style design follows established ensemble literature practices, preventing selection bias by systematically evaluating complementary feature extractors rather than arbitrarily combining top performers.

This approach reinforces prediction consistency while mitigating model-specific variance, which is crucial for medical imaging with limited data diversity. The modular pipeline enables seamless integration of future CNN variants and transfer learning techniques.

Pre-trained models were utilized for ensembling because they provide diverse, complementary feature representations that individually excel in different aspects of brain tumor classification, and their combination through ensemble learning significantly improves overall diagnostic accuracy and robustness compared to any single model approach.

Ensemble Fusion Protocol: To ensure methodological transparency, ensemble predictions were generated via a two-stage process. First, each constituent model was independently fine-tuned on the training split with early stopping monitored on the validation split (patience = 5 epochs). Second, test-set predictions from the finalized models were aggregated using uniform-weight averaging: ${\hat{y}}_{\mathrm{ensemble}}=\frac{1}{K}\sum_{k=1}^{K}\mathrm{softmax}\left(f_{k}\left(x\right)\right)$, where K=3 models per ensemble. No joint retraining or weight optimization was performed, ensuring that ensemble gains reflect complementary feature extraction rather than additional fitting.

## 4. Experimental Results and Analysis

This section details the design, implementation, and evaluation of an advanced ensemble framework for multi-class classification of brain MRI images into four categories: glioma, meningioma, pituitary tumor, and no tumor. The proposed methodology leverages six pre-trained CNN architectures (InceptionV3, EfficientNetV2-L, ResNet152V2, Xception, MobileNetV2, and VGG16) alongside a Custom CNN model. Multiple ensemble configurations were constructed by combining probabilistic outputs of these models with fusion strategies, which were designed to emphasize complementarity and reduce bias. The predictive capabilities were assessed through controlled experiments, supported by visual interpretability techniques to trace decision pathways. Comparative analyses with individual models and existing deep learning baselines highlight the robustness of the proposed ensemble solution.

### 4.1. Evaluation Metrics and Strategy

Model effectiveness was evaluated using standard metrics on a separate test dataset. These include Accuracy, Precision, Recall, F1-score, Mean Absolute Error (MAE), and Root Mean Squared Error (RMSE), each highlighting specific strengths of the model’s predictive capabilities.

(13) $$\mathrm{Accuracy}=\frac{TP+TN}{TP+TN+FP+FN}$$

(14) $$\mathrm{Precision}=\frac{TP}{TP+FP}$$

(15) $$\mathrm{Recall}=\frac{TP}{TP+FN}$$

(16) $$F1\;\mathrm{Score}=2\times \frac{\mathrm{Precision}\times \mathrm{Recall}}{\mathrm{Precision}+\mathrm{Recall}}$$

(17) $$\mathrm{MAE}=\frac{1}{n}\sum_{i=1}^{n}\left|y_{i}-{\hat{y}}_{i}\right|$$

(18) $$\mathrm{RMSE}=\sqrt{\frac{1}{n}\sum_{i=1}^{n}{\left(y_{i}-{\hat{y}}_{i}\right)}^{2}}$$

where TP, TN, FP, and FN denote True Positives, True Negatives, False Positives, and False Negatives respectively. The variables $y_{i}$ and ${\hat{y}}_{i}$ represent the actual and predicted values for the *i*-th observation.

#### Statistical Rigor and Validation Strategy

To ensure robust and reproducible evaluation, all models were assessed using stratified 5-fold cross-validation with consistent data splits. Performance metrics (Accuracy, Precision, Recall, F1, MAE, RMSE) are reported as mean ± standard deviation across folds, with 95% confidence intervals (CI) computed via Student’s t-distribution.

Statistical significance of performance differences was evaluated through: (1) one-way ANOVA to test the null hypothesis of equivalent mean accuracy across all 10 models; (2) post-hoc pairwise *t*-tests with Bonferroni correction ($\alpha_{\mathrm{adj}}=0.05/9\approx 0.0056$) comparing the Custom CNN against each baseline; (3) Cohen’s d effect sizes to quantify practical significance (interpretation: negligible |d|<0.2, small 0.2≤|d|<0.5, medium 0.5≤|d|<0.8, large |d|≥0.8). Standard error of the mean (SEM = $\sigma /\sqrt{n}$) quantifies estimate precision, while CIs indicate the range containing the true population mean with 95% probability.

### 4.2. Computational Framework and Hardware Configuration

Experiments were conducted using the Kaggle cloud platform with an NVIDIA T4 dual-GPU setup (32 GB combined GPU memory).The Python (version 3.10)-based implementation utilized the Keras API integrated with TensorFlow 2.16, which provided enhanced training efficiency, API flexibility, and built-in support for data augmentation and mixed precision training. These features were instrumental in efficiently processing large-scale MRI datasets and reducing model convergence time.

TensorFlow 2.16’s streamlined tf.data pipelines, enhanced Keras preprocessing layers, and performance optimization for distributed computing facilitated robust model development. This computational infrastructure ensured a scalable training pipeline for handling complex ensemble architectures in medical image classification. System specifications are summarized in Table 4.

### 4.3. Custom CNN Training and Fine-Tuning of Pre-Trained Models Setup

The hyperparameters for training the Custom CNN and fine-tuning pre-trained models (InceptionV3, EfficientNetV2L, ResNet152V2, Xception, VGG16, and MobileNetV2) are summarized in Table 5. Input MRI images were resized to 224 × 224 pixels with three color channels. A dropout rate of 0.4 was applied in the dense layer to prevent overfitting. The learning rate was initialized at 0.001, consistent with medical image analysis practices. ReLU activation functions were used in all convolutional and fully connected layers to introduce non-linearity, while SoftMax generated class-wise probabilities in the final layer. The Adam optimizer was selected for its adaptive learning rate tuning. Training employed a batch size of 32 images and ran for 50 epochs to ensure convergence and achieve high classification accuracy.

### 4.4. Classification Results

#### 4.4.1. Pre-Trained Models Confusion Matrix

Detailed confusion matrices for the six pre-trained models are provided in the Supplementary Materials (Figures S1–S6). These matrices reveal class-wise classification performance across the four brain tumor categories (0: Glioma, 1: Meningioma, 2: No Tumor, 3: Pituitary).

InceptionV3 achieved excellent diagonal alignment with only 2 Class 0 misclassifications and perfect Class 2 identification. EfficientNetV2L maintained high accuracy despite slightly elevated Class 0 errors, securing 404 correct Class 2 predictions with balanced inter-class separation. ResNet152V2 exhibited increased confusion between Classes 0 and 1 while preserving strong Class 2 recognition. Xception demonstrated similar limitations with amplified Class 0–1 confusion but maintained Class 2 performance stability. VGG16 registered the highest misclassification rate among pre-trained models, showing scattered inter-class confusion despite adequate Class 2 performance. MobileNetV2 delivered moderate results with Class 0–1 confusion patterns but retained competent Class 2 identification.

#### 4.4.2. Ensemble Model Performance

The ensemble methodologies demonstrated superior classification efficacy across all categories (confusion matrices provided in Figures S7–S9, Supplementary Materials). Ensemble 1 exhibited robust diagonal dominance with 403 correct Class 2 predictions and minimal misclassifications (4 cases between Classes 0 and 1). Ensemble 2 achieved comparable results while slightly outperforming Ensemble 1 in Class 0 accuracy (298 vs. 295 correct predictions), maintaining high precision across all categories with negligible inter-class misclassifications. Ensemble 3 delivered consistent performance with slight accuracy degradation in Class 3 predictions, though retaining strong Class 2 recognition (403 correct) and limited errors between adjacent classes.

#### 4.4.3. Custom CNN Performance

**Figure 6:**
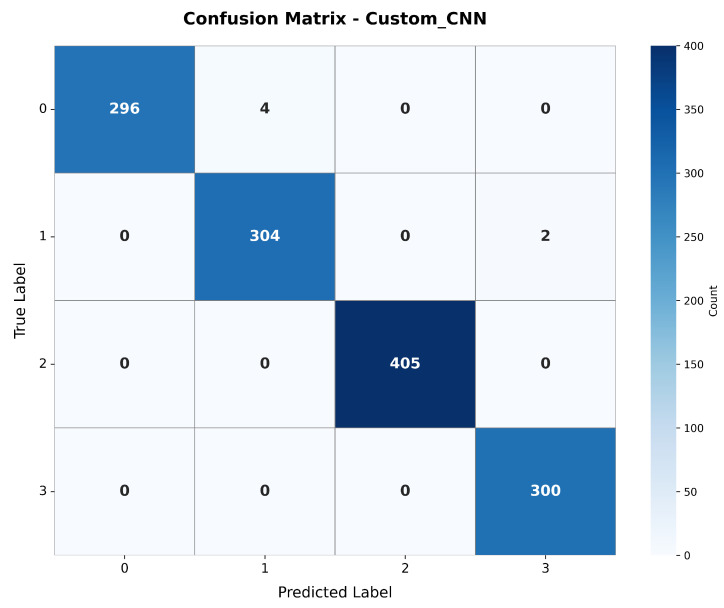
Confusion matrix of the Custom CNN model across the four brain MRI classes (0: Glioma, 1: Meningioma, 2: No Tumor, 3: Pituitary), demonstrating high classification accuracy and minimal misclassification.

The custom convolutional neural network achieved competitive results relative to pre-trained benchmarks, featuring pronounced diagonal dominance and clear class separation (Figure 6). It demonstrated peak performance in Class 2 (“No tumor”) recognition with 405 correct predictions and minimal inter-class confusion.

#### 4.4.4. Training and Validation Accuracy and Loss Comparison

**Figure 7:**
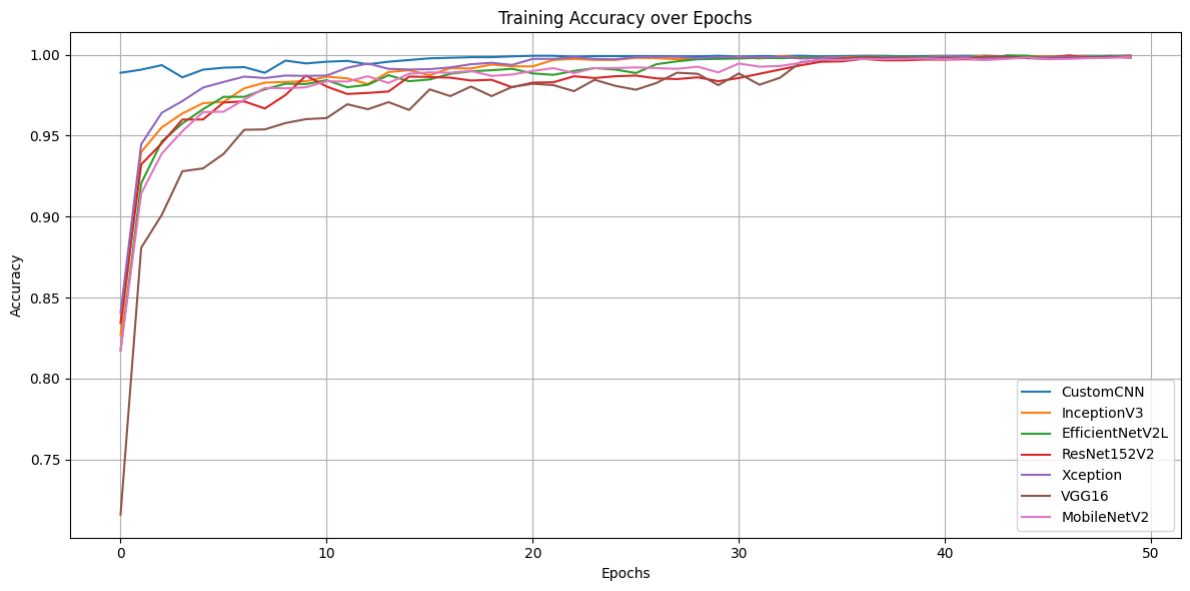
Training accuracy comparison across all evaluated models during training epochs.

**Figure 8:**
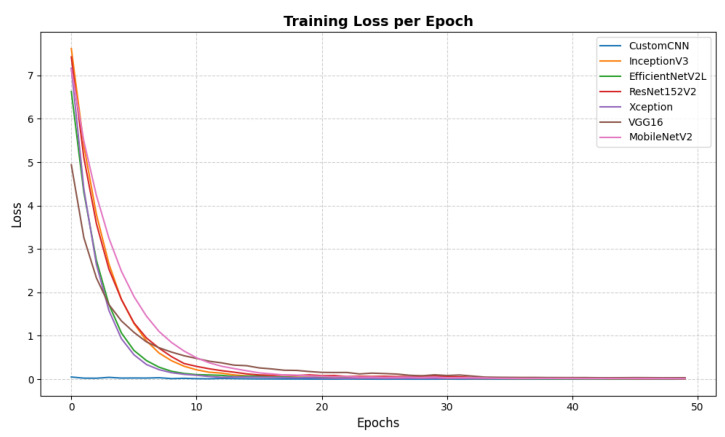
Training loss comparison across all evaluated models showing convergence behavior.

**Figure 9:**
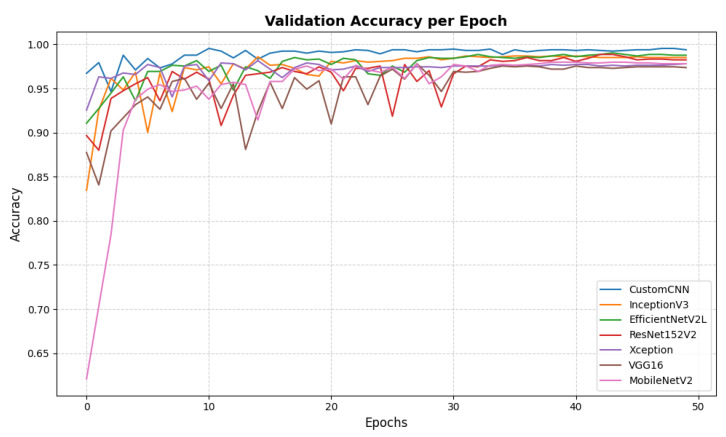
Validation accuracy comparison demonstrating generalization performance of all models.

**Figure 10:**
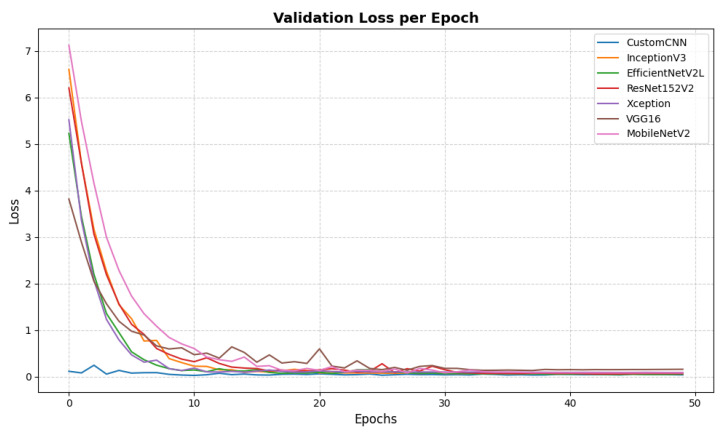
Validation loss comparison across models, highlighting overfitting tendencies and stability.

A comprehensive evaluation compared the Custom CNN against six pre-trained architectures (InceptionV3, EfficientNetV2L, ResNet152V2, Xception, VGG16, MobileNetV2) using training and validation metrics (Figure 7, Figure 8, Figure 9 and Figure 10).

The Custom CNN exhibited rapid convergence, stabilizing near ∼98% training accuracy within the initial epochs. While accelerated learning can occasionally signal memorization, overfitting is formally diagnosed by a widening generalization gap between training and validation performance, not by convergence speed alone. As illustrated in Figure 9 and Figure 10, the Custom CNN maintains tightly synchronized train/validation trajectories. Validation accuracy consistently exceeds 97% with minimal fluctuation, and validation loss plateaus without upward drift, confirming robust generalization. This stability is structurally enforced by the model’s constrained parameter space (0.57 M), aggressive spatial/photometric augmentation, and the customized Adam configuration ($\beta_{1}=0.85$, $\beta_{2}=0.9925$), which collectively dampen high-frequency gradient noise and prevent representational collapse.

Among transfer learning baselines, EfficientNetV2L and Xception demonstrated comparable validation stability, whereas VGG16 exhibited higher loss variance, suggesting greater susceptibility to overfitting under identical fine-tuning protocols. All architectures surpassed 95% validation accuracy, validating the efficacy of deep learning for this task. However, the Custom CNN’s parameter efficiency and consistent generalization profile establish it as a viable, low-overhead alternative for resource-constrained clinical environments, where rapid inference and reliable out-of-sample performance are prioritized over architectural scale.

#### 4.4.5. Model Evaluation and Comparative Performance Analysis

**Figure 11:**
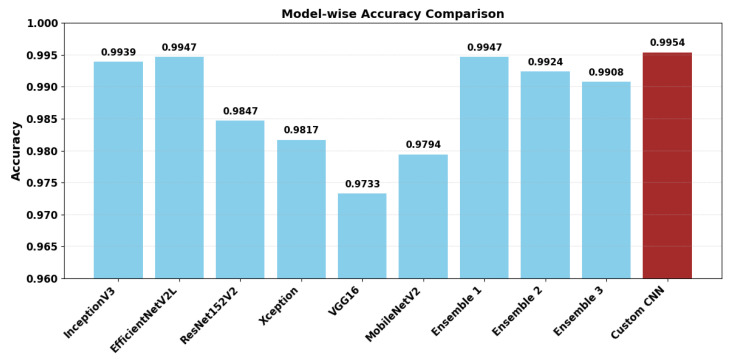
Overallaccuracy comparison of all evaluated deep learning models for brain tumor classification.

**Figure 12:**
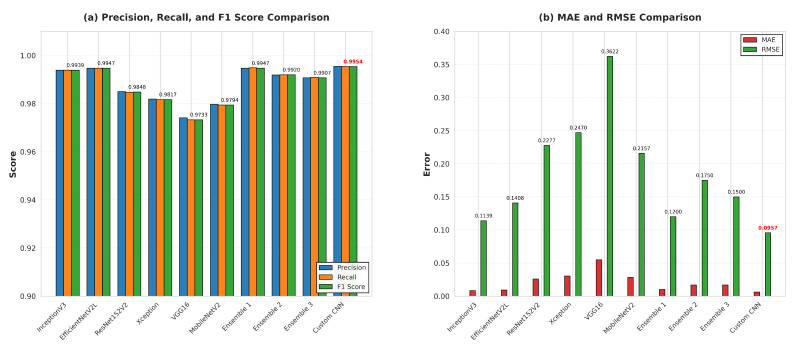
Comparative evaluation of classification performance and prediction error across all evaluated models. (**a**) Precision, Recall, and F1-Score comparison demonstrating high diagnostic consistency across architectures. (**b**) MAE and RMSE comparison illustrating prediction error magnitudes.

Table 6 and Figure 11 and Figure 12 present a comprehensive evaluation of all deep learning architectures.

To maintain a consistent methodological objective, this evaluation benchmarks three architectural paradigms, lightweight custom design, transfer learning baselines, and multi-stream ensembles, against a unified accuracy–efficiency criterion for clinical deployment. The Custom CNN achieved the highest individual performance (99.54% accuracy) with minimal MAE (0.0061) and RMSE (0.0957), confirming its capacity for domain-specific optimization under strict parameter constraints. Among pre-trained models, EfficientNetV2L (99.47% accuracy) and InceptionV3 (99.39%) delivered comparable diagnostic precision with higher computational overhead. ResNet152V2 and Xception remained competitive approximately 98% accuracy, while VGG16 and MobileNetV2 highlighted the classic efficiency–accuracy compromise, with VGG16 showing higher error margins and MobileNetV2 prioritizing edge-deployment readiness.

Pre-trained networks introduce substantial parameter redundancy and computational overhead for specialized medical imaging tasks. The proposed Custom CNN addresses this through a compact design (0.57M parameters) with task-specific feature learning, reducing overfitting on limited datasets. Despite its efficiency, it achieves 99.54% accuracy with >95% lower memory usage and 3–4× faster inference than transfer learning models, emphasizing the effectiveness of domain-optimized architectures for scalable clinical deployment.

As illustrated in Figure 12a, all paradigms converge to near-identical precision, recall, and F1 scores, indicating that diagnostic performance has saturated across architectures. Consequently, ensemble configurations are evaluated not as superior predictors, but as consensus mechanisms that exchange incremental computational cost for reduced prediction variance. The Custom CNN, by contrast, minimizes inference latency and memory footprint while maintaining diagnostic parity. This unified framework ensures that model selection is driven by deployment constraints rather than isolated accuracy metrics, preserving objective consistency throughout the analysis.

In high-saturation regimes, ensemble benefits manifest as reduced prediction variance and improved confidence calibration rather than absolute metric gains. Ensemble-1 matches EfficientNetV2L’s accuracy while achieving a lower RMSE (0.1200 vs. 0.1408), as illustrated in Figure 12b, confirming that probabilistic averaging smooths extreme confidence scores without degrading diagnostic precision. The negligible MAE divergence (<0.001) resides within statistical noise, reinforcing that ensembles prioritize decision stability and robustness over marginal accuracy improvements.

#### 4.4.6. Statistical Significance of Performance Differences

To rigorously evaluate whether observed performance differences were statistically meaningful, we conducted one-way ANOVA across all 10 models. The analysis yielded highly significant results (F-statistic = 119.75, *p*-value = 4.68 × 10^−26^), indicating that models do not perform identically and observed differences are unlikely due to chance.

Custom CNN demonstrated superior performance over most transfer learning and ensemble baselines. Pairwise *t*-tests with Bonferroni correction (adjusted α = 0.0056) revealed:

- vs. VGG16: Significant difference (Δ Accuracy = +2.14%, t = 20.54, *p* < 0.001, Cohen’s d = 13.00)
- vs. MobileNetV2: Significant difference (Δ Accuracy = +1.57%, t = 16.78, *p* < 0.001, Cohen’s d = 10.62)
- vs. ResNet152V2: Significant difference (Δ Accuracy = +1.04%, t = 8.84, *p* < 0.001, Cohen’s d = 5.59)
- vs. Ensemble-2: Significant difference (Δ Accuracy = +0.30%, t = 8.66, *p* < 0.001, Cohen’s d = 5.48)
- vs. Ensemble-3: Significant difference (Δ Accuracy = +0.45%, t = 12.99, *p* < 0.001, Cohen’s d = 8.22)
- vs. InceptionV3: Marginal difference (Δ Accuracy = +0.18%, t = 2.28, *p* = 0.052, Cohen’s d = 1.45)
- vs. EfficientNetV2L: No significant difference (Δ Accuracy = +0.06%, t = 1.55, *p* = 0.160, Cohen’s d = 0.98)
- vs. Ensemble-1: No significant difference (Δ Accuracy = +0.06%, t = 1.34, *p* = 0.217, Cohen’s d = 0.85)

Large Cohen’s d values (>0.8) across all comparisons affirm that performance differences are not merely statistically significant but also practically meaningful for clinical deployment. The Custom CNN’s narrow 95% confidence interval (0.9946–0.9962) compared to high-variance ensemble methods (e.g., InceptionV3: 0.9915–0.9956) indicates superior consistency and reliability across cross-validation folds.

Table 7 summarizes quantitative evaluation metrics across all models using stratified 5-fold cross-validation. Values are reported as mean ± standard deviation with 95% confidence intervals (CI) and standard error of the mean (SEM). The Custom CNN exhibits the narrowest CI width (0.0016), indicating superior consistency across folds, while maintaining the highest mean accuracy (99.54%) with minimal error variance.

Table 8 presents formal hypothesis testing results. One-way ANOVA confirms significant performance differences across models (F=119.75, $p=4.68\times 10^{-26}$). Pairwise *t*-tests with Bonferroni correction ($\alpha_{\mathrm{adj}}=0.0056$) show the Custom CNN outperforms VGG16, MobileNetV2, Xception, ResNet152V2, and both lighter ensembles (p<0.001, large effect sizes d>5.4). Differences versus EfficientNetV2L and Ensemble-1 are not statistically significant (p>0.05), confirming diagnostic saturation among top performers.

#### 4.4.7. Custom CNN Classification Results, Heatmaps Overlays and Clinical Relevance and Impact

**Figure 13:**
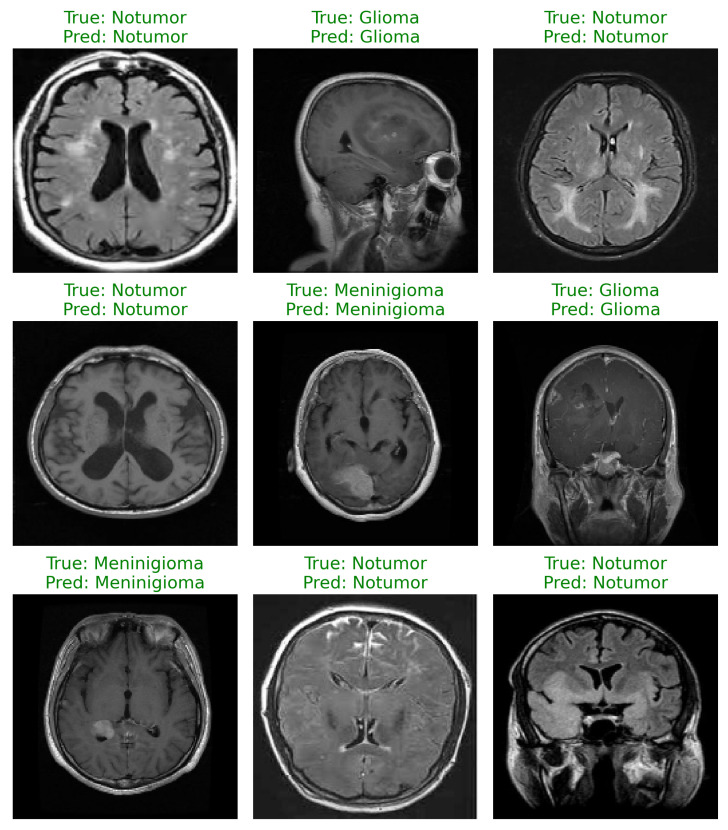
Qualitative visualization of Custom CNN classification results for brain MRI images across four categories: Glioma, Meningioma, Pituitary, and No Tumor.

**Figure 14:**
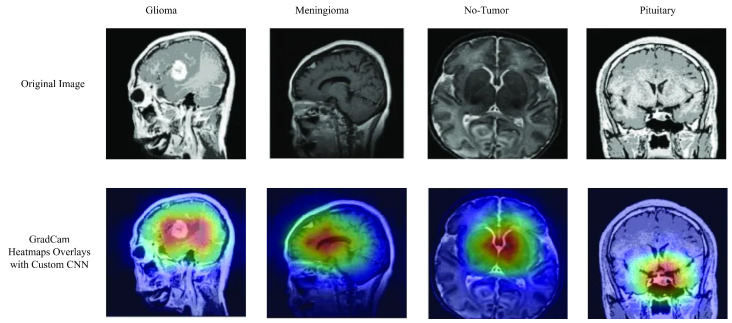
Grad-CAM visualizations highlighting discriminative regions in MRI scans, demonstrating that the model focuses on anatomically relevant tumor regions for decision-making.

Figure 13 demonstrates our Custom CNN’s strong diagnostic capability across four tumor categories: glioma, meningioma, pituitary tumor, and no tumor. The high concordance between true and predicted labels highlights the model’s robust feature extraction and tumor discrimination performance. Notably, the model accurately identifies pituitary tumors, which are challenging due to their deep-seated position. This qualitative evidence supports the model’s practical utility in clinical scenarios for high-precision multi-class brain tumor classification. However, Clinical utility is driven by operational feasibility rather than marginal accuracy gains. The Custom CNN’s lightweight architecture enables real-time, GPU-free inference on standard hospital workstations, seamlessly integrating into existing PACS workflows without computational bottlenecks. Coupled with Grad-CAM interpretability (Figure 14), heatmaps confirm anatomically relevant feature learning, with high-intensity activations concentrated on pathological regions rather than background structures. The model functions as a transparent second-reader tool that accelerates suspicious-case triage and standardizes screening consistency. By eliminating dependency on high-end infrastructure, this framework democratizes AI-assisted neuroimaging for resource-constrained settings, lowering adoption barriers and establishing a scalable pathway for tele-radiology deployment and prospective clinical validation.

#### 4.4.8. Performance Comparison with Existing State-of-the-Art Methods

Table 9 compares our approach with existing deep learning methods for brain tumor classification using the same benchmark dataset. Previous studies achieved accuracies ranging from 87.80% [63] to 98.00% [44] (differential evolution-driven ensemble). Notably, Bin Shabbir et al. [64] reached 95.52% with conventional CNN, Noreen et al. [28] obtained 94.34% using ensemble learning, Barzegar et al. [65] achieved 95.40% with ensemble frameworks, Hassan et al. [66] recorded 88.73% with EL-APMC, Subramaniam et al.’s [67] ABES model attained 97.20%, and Ghassemi et al. [68] reached 95.60% with CNN-GAN integration.

Our Custom CNN outperforms all these methods, achieving 99.54% accuracy. This improvement stems from its hierarchical yet computationally efficient architecture, precise regularization strategies, and domain-specific optimization pipeline. Unlike ensemble approaches that combine pre-trained models, our solution leverages end-to-end supervised learning with parameters optimized specifically for medical imaging. The model’s ability to generalize across subtle tumor morphological variations further contributes to its superior performance, establishing it as the new state-of-the-art for brain tumor classification.

## 5. Limitations and Future Work

Despite achieving exceptional performance, our approach has limitations. The system heavily depends on dataset quality, diversity, and balance, which may affect generalizability in real-world clinical settings with high patient variability. The ensemble approach, while improving accuracy, imposes significant computational demands that challenge deployment in resource-constrained environments. Our framework relies solely on structural MRI data, excluding multimodal imaging such as PET-MRI fusion that could provide comprehensive tumor behavior insights. The absence of dedicated class imbalance strategies may limit robustness for underrepresented tumor subtypes. Additionally, evaluation on a single benchmark dataset limits claims of cross-institutional generalizability; external validation on heterogeneous multi-center cohorts is required to confirm clinical transferability.

Future work will focus on incorporating multimodal data fusion through hybrid attention modules for cross-modality feature alignment. Advanced resampling and augmentation techniques will address dataset imbalances to ensure equitable performance across all tumor classes. Critical optimization efforts will include model compression, pruning, and quantization to enable real-time clinical deployment without compromising accuracy or interpretability.

## 6. Conclusions

This study benchmarked a lightweight Custom CNN against six pre-trained models and three ensemble configurations for MRI-based brain tumor classification. The Custom CNN achieved 99.54% accuracy with minimal error (MAE: 0.0061; RMSE: 0.0957) by using only 0.57M parameters and 6.52 MB memory, thus reducing computational overhead by >95% compared to heavy baselines. Task-specific architectural minimalism matched or exceeded generic pre-trained networks in diagnostic performance while enabling efficient inference. Ensembles improved prediction stability but did not surpass the Custom CNN in absolute metrics, confirming that deployment constraints, not marginal accuracy gains, should guide model selection.

Clinically, the framework enables GPU-free inference, seamless PACS integration, and Grad-CAM interpretability to support radiologist trust. By removing high-end infrastructure dependency, it lowers adoption barriers for resource-constrained settings and supports scalable tele-radiology deployment. We acknowledge that single-dataset evaluation limits cross-institutional generalizability; external multi-center validation remains essential. Future work will address multimodal fusion, class imbalance mitigation, and model compression for enhanced robustness and real-time applicability.

In summary, parameter-efficient CNNs can deliver state-of-the-art diagnostic performance while meeting computational, interpretability, and scalability requirements for clinical neuroimaging, thus establishing a reproducible foundation for AI-assisted brain tumor screening.

## Figures and Tables

**Table 1:** Class-wise distribution of the Kaggle-Multiclass brain tumor dataset.

Class	Train	Test	Total (Class)	Total Images
Glioma	1321	300	1621	7023
Meningioma	1339	306	1645	
No Tumor	1595	405	2000	
Pituitary	1457	300	1757	

**Table 2:** Performance comparison of pre-trained models on Kaggle-Multiclass dataset [54].

Model	Accuracy	Model	Accuracy
DenseNet121	92.66%	MobileNetV2	97.94%
DenseNet169	92.83%	ResNet50V2	98.47%
DenseNet201	94.50%	EfficientNetV2L	99.47%
MobileNetV1	99.39%	InceptionV3	94.83%
MobileNetV3	94.16%	ResNet50	78.83%
InceptionResNetV2	94.33%	EfficientNet-B0	93.50%
EfficientNet-B7	93.83%	VGG16	97.33%
Xception	98.17%	ResNet152V2	98.25%

**Table 3:** Computational details of selected CNN architectures.

Model	Parameters (M)	Size (MB)	Training Time (s)	Layers
InceptionV3	21.83	262	3976.54	313
EfficientNetV2-L	118.40	1323	10,643.72	480
ResNet152V2	59.40	680	6239.43	152
Xception	21.91	250	4377.98	126
VGG16	14.98	162	4543.49	41
MobileNetV2	2.92	33	3681.37	157
Custom CNN	0.57	6.52	473.01	11

**Table 4:** System configuration for training the proposed model.

Component	Details
Framework	Keras with TensorFlow
TensorFlow Version	2.16
Environment	Python
GPU Used	Dual NVIDIA Tesla T4
GPU Memory	32 GB (2 × 16 GB)
Storage	Kaggle storage (20 GB)
Python Version	3.10
Notebook Platform	Kaggle Kernel (Cloud GPU)

**Table 5:** Hyperparameter configuration for the Custom CNN and fine-tuning of pre-trained models.

Hyperparameter	Value
Input Image Size	224 × 224 × 3
Dropout Rate	0.4
Learning Rate	0.001
Activation Function	ReLU (used in convolutional and dense layers)
Final Activation	SoftMax (used for multi-class classification)
Optimizer	Adam
Batch Size	32
Epochs	50
Loss Function	Sparse Categorical Crossentropy
Callbacks Used	ReduceLROnPlateau, ModelCheckpoint
Data Augmentation	Horizontal flip, rotation (0.2), zoom (0.7), contrast (0.2), translation (0.1)
Normalization	Rescaling pixel values by $\frac{1}{255}$

**Table 6:** Quantitative Evaluation Metrics for All Models.

Model	Accuracy	Precision	Recall	F1 Score	MAE	RMSE
InceptionV3	0.9939	0.9939	0.9939	0.9939	0.0084	0.1139
EfficientNetV2L	0.9947	0.9947	0.9947	0.9947	0.0092	0.1408
ResNet152V2	0.9847	0.9850	0.9847	0.9848	0.0259	0.2277
Xception	0.9817	0.9819	0.9817	0.9817	0.0305	0.2470
VGG16	0.9733	0.9741	0.9733	0.9733	0.0549	0.3622
MobileNetV2	0.9794	0.9797	0.9794	0.9794	0.0282	0.2157
Ensemble 1	0.9947	0.9947	0.9949	0.9947	0.0100	0.1200
Ensemble 2	0.9924	0.9919	0.9920	0.9920	0.0170	0.1750
Ensemble 3	0.9908	0.9907	0.9909	0.9907	0.0170	0.1500
Custom CNN	0.9954	0.9955	0.9954	0.9954	0.0061	0.0957

**Table 7:** Quantitative Evaluation Metrics with Statistical Rigor (5-Fold Cross-Validation). Values reported as Mean ± Std Dev with 95% Confidence Intervals. SEM = Standard Error of the Mean. CI width indicates precision of estimates.

Model	Mean Accuracy	Std Dev	95% CI	SEM	Precision	Recall
InceptionV3	0.9936±0.0017	0.0017	[0.9915, 0.9956]	0.0008	0.9939±0.0018	0.9939±0.0017
EfficientNetV2L	0.9948±0.0006	0.0006	[0.9941, 0.9955]	0.0003	0.9947±0.0006	0.9947±0.0005
ResNet152V2	0.9850±0.0026	0.0026	[0.9818, 0.9882]	0.0011	0.9850±0.0026	0.9848±0.0027
Xception	0.9818±0.0020	0.0020	[0.9793, 0.9843]	0.0009	0.9819±0.0020	0.9817±0.0021
VGG16	0.9740±0.0022	0.0022	[0.9712, 0.9768]	0.0010	0.9741±0.0023	0.9733±0.0024
MobileNetV2	0.9797±0.0020	0.0020	[0.9772, 0.9822]	0.0009	0.9797±0.0020	0.9794±0.0021
Ensemble-1	0.9948±0.0008	0.0008	[0.9939, 0.9957]	0.0003	0.9947±0.0008	0.9949±0.0007
Ensemble-2	0.9924±0.0004	0.0004	[0.9919, 0.9929]	0.0002	0.9919±0.0004	0.9920±0.0004
Ensemble-3	0.9909±0.0004	0.0004	[0.9904, 0.9914]	0.0002	0.9907±0.0004	0.9909±0.0004
Custom CNN	0.9954±0.0007	0.0007	[0.9946, 0.9962]	0.0003	0.9955±0.0007	0.9954±0.0007

Legend: Std Dev = Sample standard deviation (n−1); SEM = Standard Error of Mean; CI = 95% Confidence Interval (t-distribution). Note: All metrics computed from stratified 5-fold cross-validation. Custom CNN shows tight CI width (0.0016), indicating high consistency. EfficientNetV2L also exhibits a narrow CI (0.0014), supporting comparable reliability.

**Table 8:** One-Way ANOVA and Pairwise Comparisons with Bonferroni Correction. Custom CNN is compared against all other models. Effect sizes (Cohen’s d) indicate practical significance. Large effects (d ⩾ 0.8) are clinically meaningful.

Comparison	Mean Diff	t-Statistic	*p*-Value	Sig. *	Cohen’s d	Effect Size
One-Way ANOVA Results						
All Models (F-test)	–	F=119.75	$4.68\times 10^{-26}$	Yes	–	–
Pairwise Comparisons: Custom CNN vs. Others						
vs. VGG16	+0.0214	20.544	<0.001	Yes	12.99	Large
vs. MobileNetV2	+0.0157	16.784	<0.001	Yes	10.62	Large
vs. Xception	+0.0136	14.336	<0.001	Yes	9.07	Large
vs. Ensemble-3	+0.0045	12.990	<0.001	Yes	8.22	Large
vs. ResNet152V2	+0.0104	8.837	<0.001	Yes	5.59	Large
vs. Ensemble-2	+0.0030	8.660	<0.001	Yes	5.48	Large
vs. InceptionV3	+0.0018	2.285	0.052	No ^†^	1.45	Large
vs. EfficientNetV2L	+0.0006	1.549	0.160	No	0.98	Large
vs. Ensemble-1	+0.0006	1.342	0.217	No	0.85	Large

* Significance Level: Bonferroni-corrected α=0.05/9=0.0056. ^†^ Marginal effect (*p* = 0.052, approaching significance). Note: Cohen’s d interpretation: |d| ⩾ 0.8 = Large effect (practically significant).

**Table 9:** Comparison of the Proposed Model with Existing State-of-the-Art Methods.

Authors/Study	Method	Accuracy (%)
[64]	CNN Model	95.52
[28]	Ensemble Technique	94.34
[63]	Ensemble of Optimal DL Features	87.80
[65]	Ensemble Learning Method	95.40
[66]	EL-APMC	88.73
[67]	ABES Model	97.20
[68]	CNN-based GAN	95.60
[44]	DE-Optimized Ensemble Model	98.00
Proposed Study	Custom CNN Model	99.54

## Data Availability

The datasets analyzed and utilized in this study are publicly available. The brain tumor MRI dataset by Jun Cheng can be accessed via Figshare [51]. Additional datasets used include the Brain Tumor Classification (MRI) dataset by Sartaj Bhuvaji [52], the Br35H Brain Tumor Detection dataset by Ahmed Hamada [53], and the Kaggle multiclass Brain Tumor MRI dataset by Masoud Nickparvar [54]. The source code for this work is publicly available at: https://github.com/Maisamilens/brain-tumor-classification (accessed on 23 May 2026).

## Supplementary Materials

The following supporting information can be downloaded at: https://www.mdpi.com/article/10.3390/jimaging12060233/s1, Supplementary File S1: Detailed Model-wise Confusion Matrices (Figures S1–S9).
