# Supplementary material for: Brain Tumor Classification in MRI Images Using Combined Transfer Learning and Convolutional Neural Networks
Source: J Imaging. 2026 May 28;12(6):233. doi: 10.3390/jimaging12060233 (PMC13301762; doi:10.3390/jimaging12060233)
Supplement: Supplementary file 1 [file jimaging-12-00233-s001.zip › jimaging-4244857-supplementary.pdf]

Supplementary File S1: Detailed Model-wise Confusion Matrices

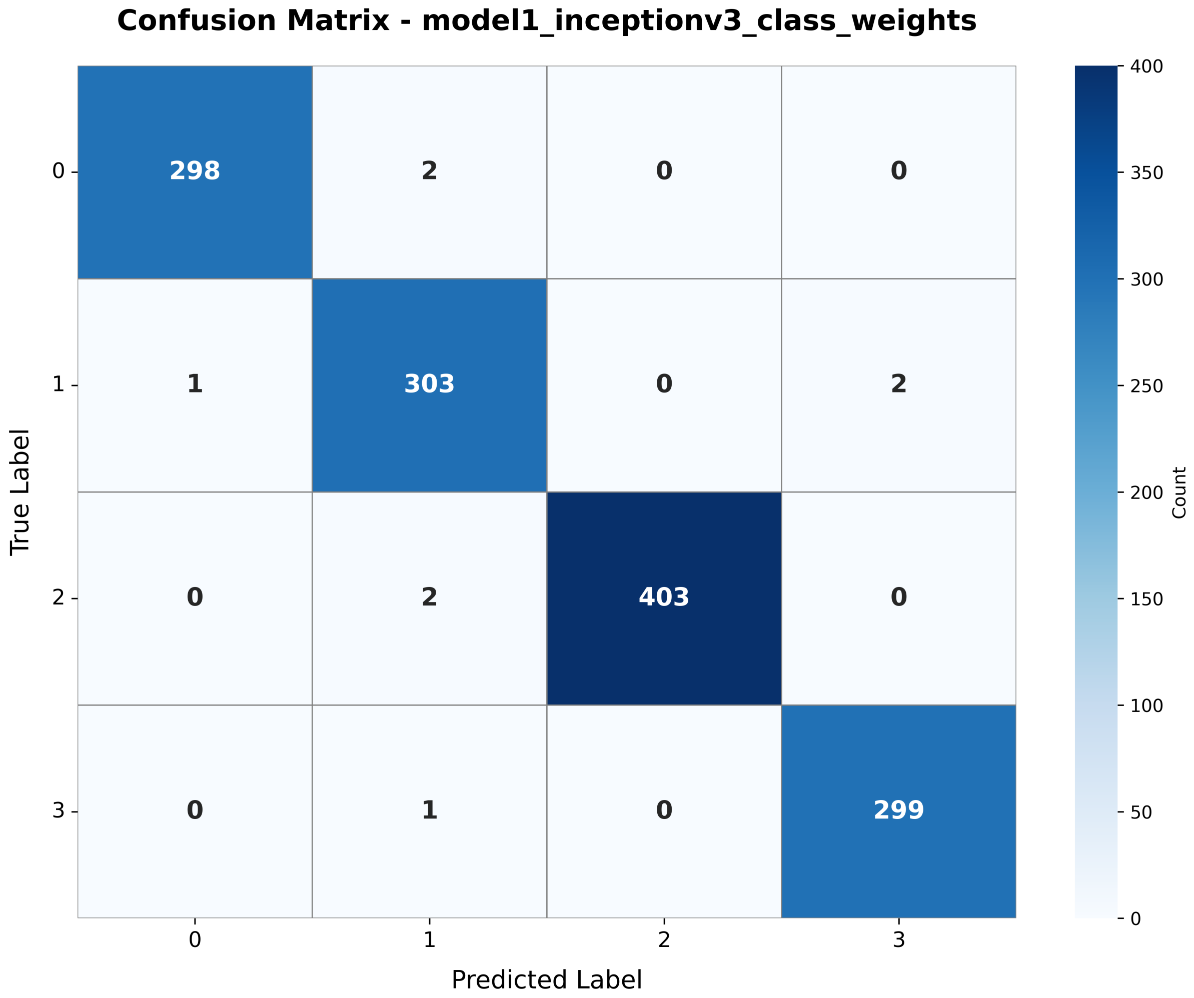

Figure S1: Confusion matrix of the InceptionV3 model showing class-wise classification performance across four brain tumor categories.

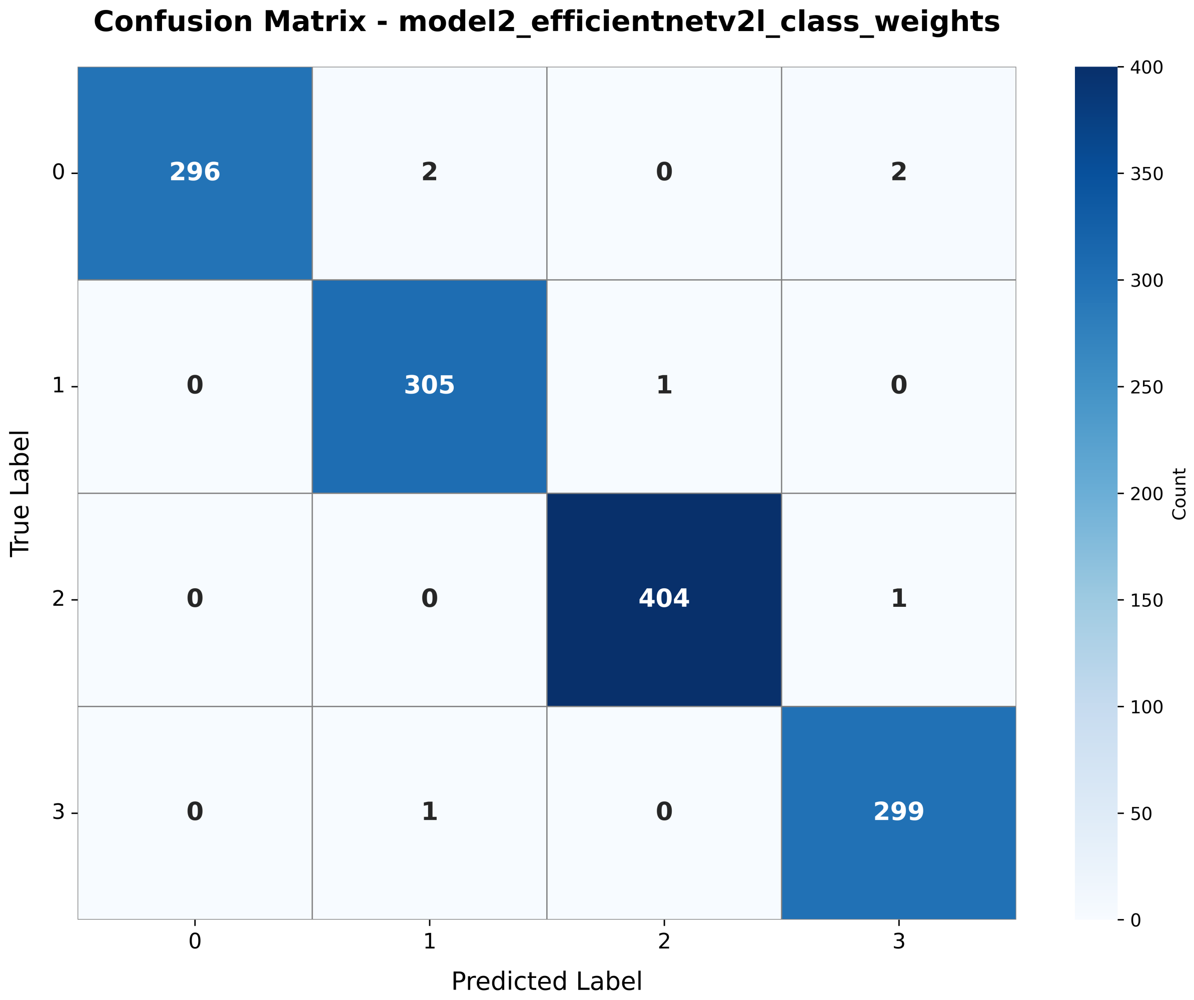

Figure S2: Confusion matrix of the EfficientNetV2L model illustrating prediction distribution across Glioma, Meningioma, Pituitary, and No Tumor classes.

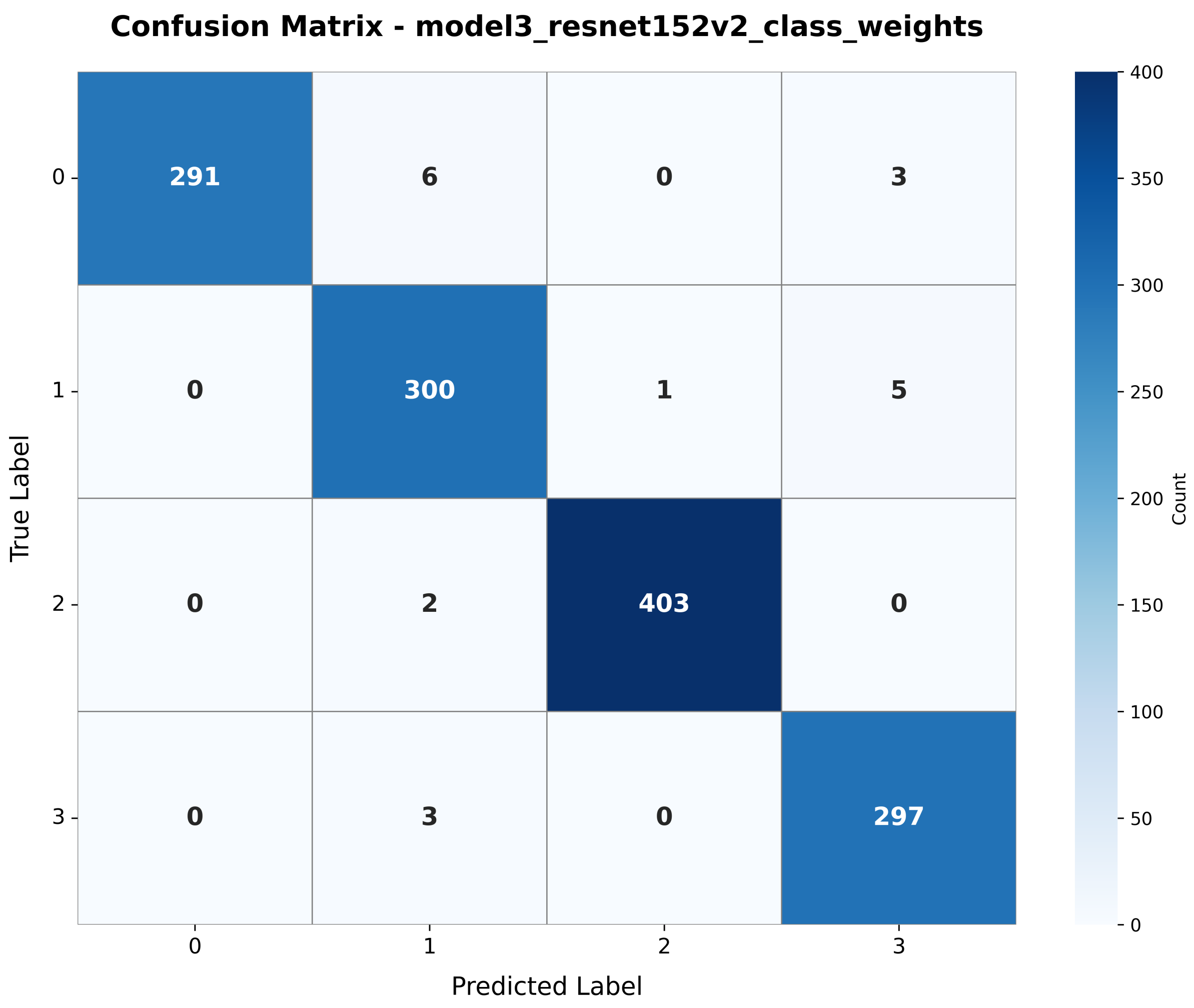

Figure S3: Confusion matrix of the ResNet152V2 model showing classification accuracy across all brain tumor categories.

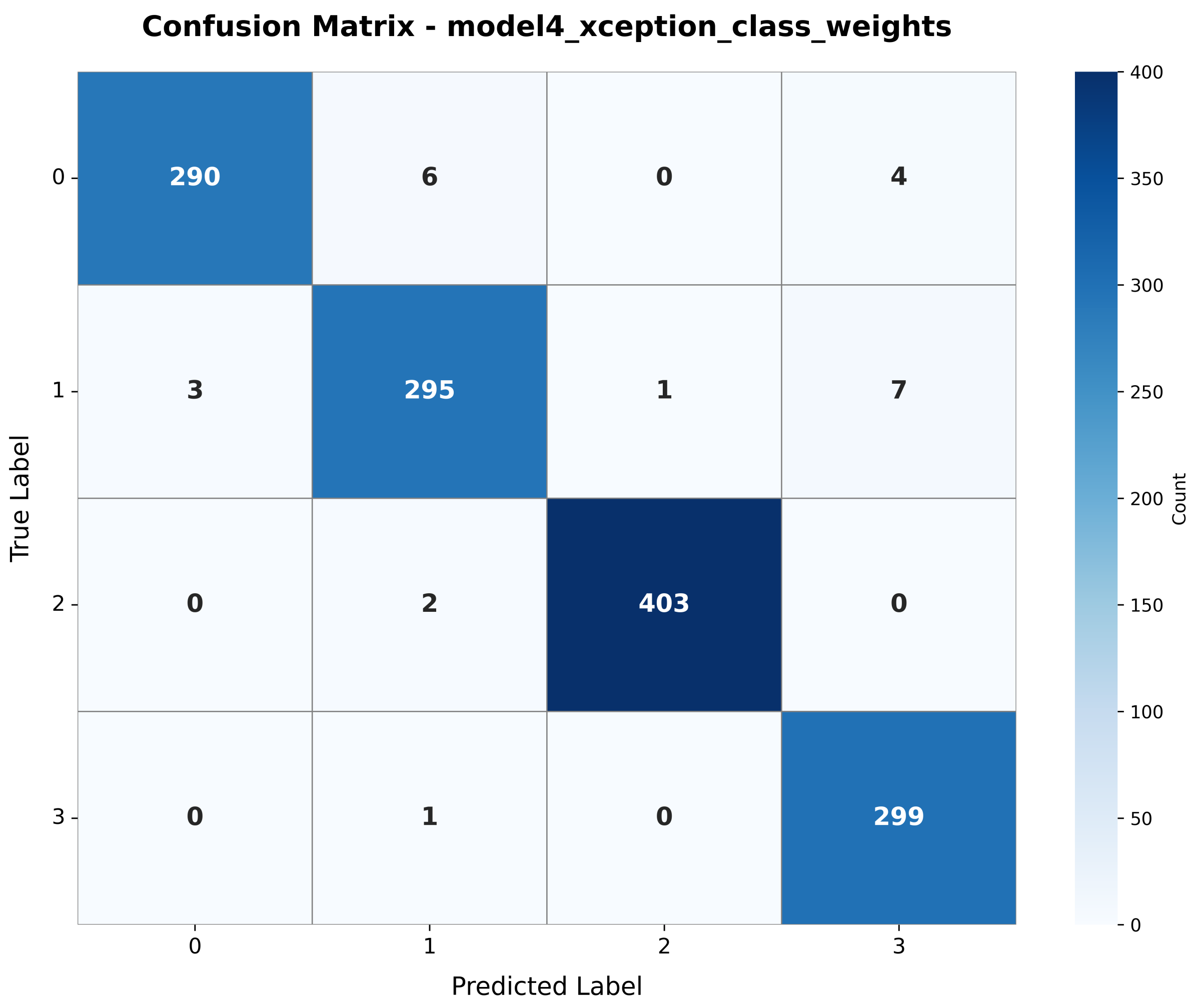

Figure S4: Confusion matrix of the Xception model presenting class-wise prediction performance for brain MRI classification.

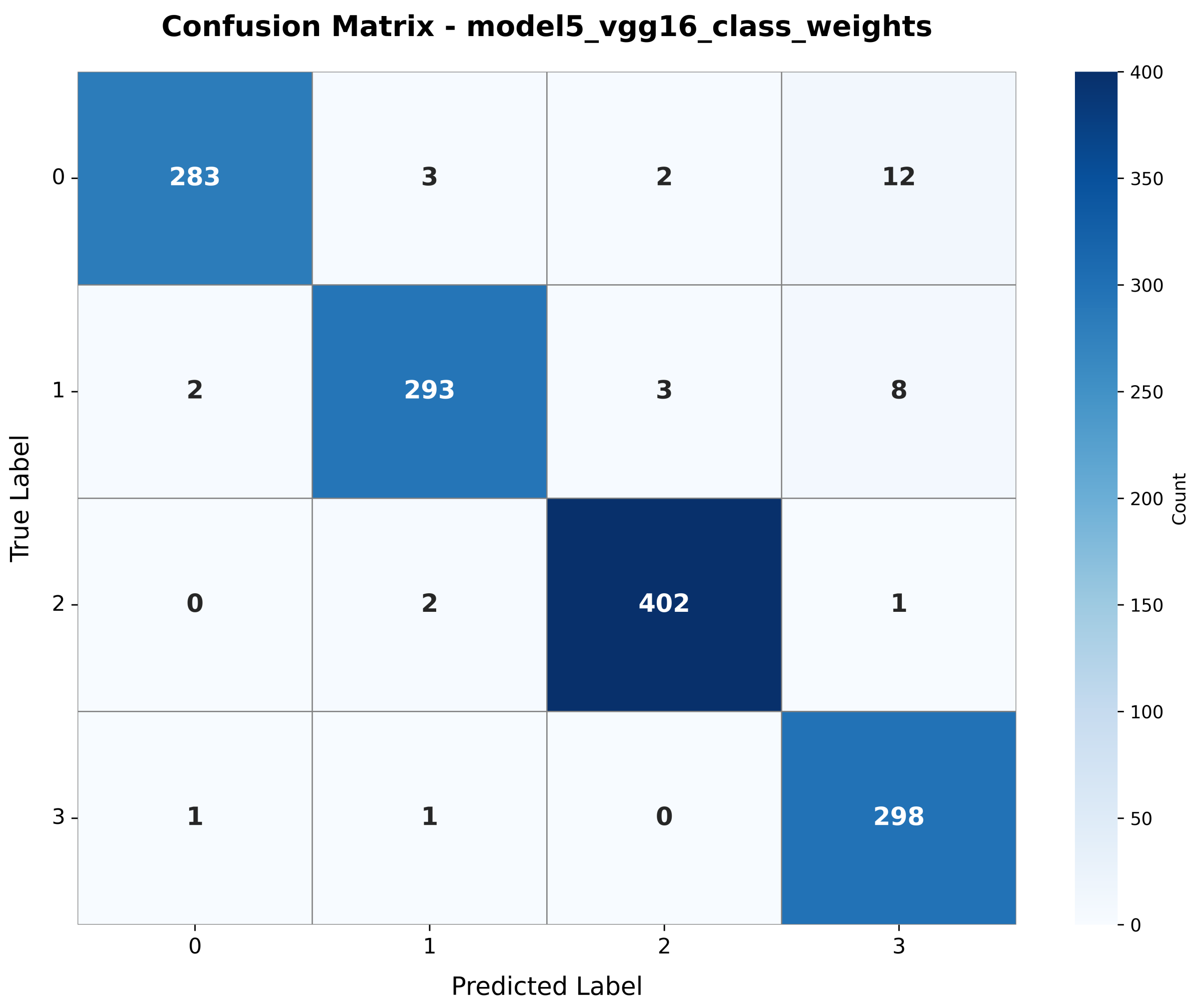

Figure S5: Confusion matrix of the VGG16 model illustrating classification results across all tumor categories.

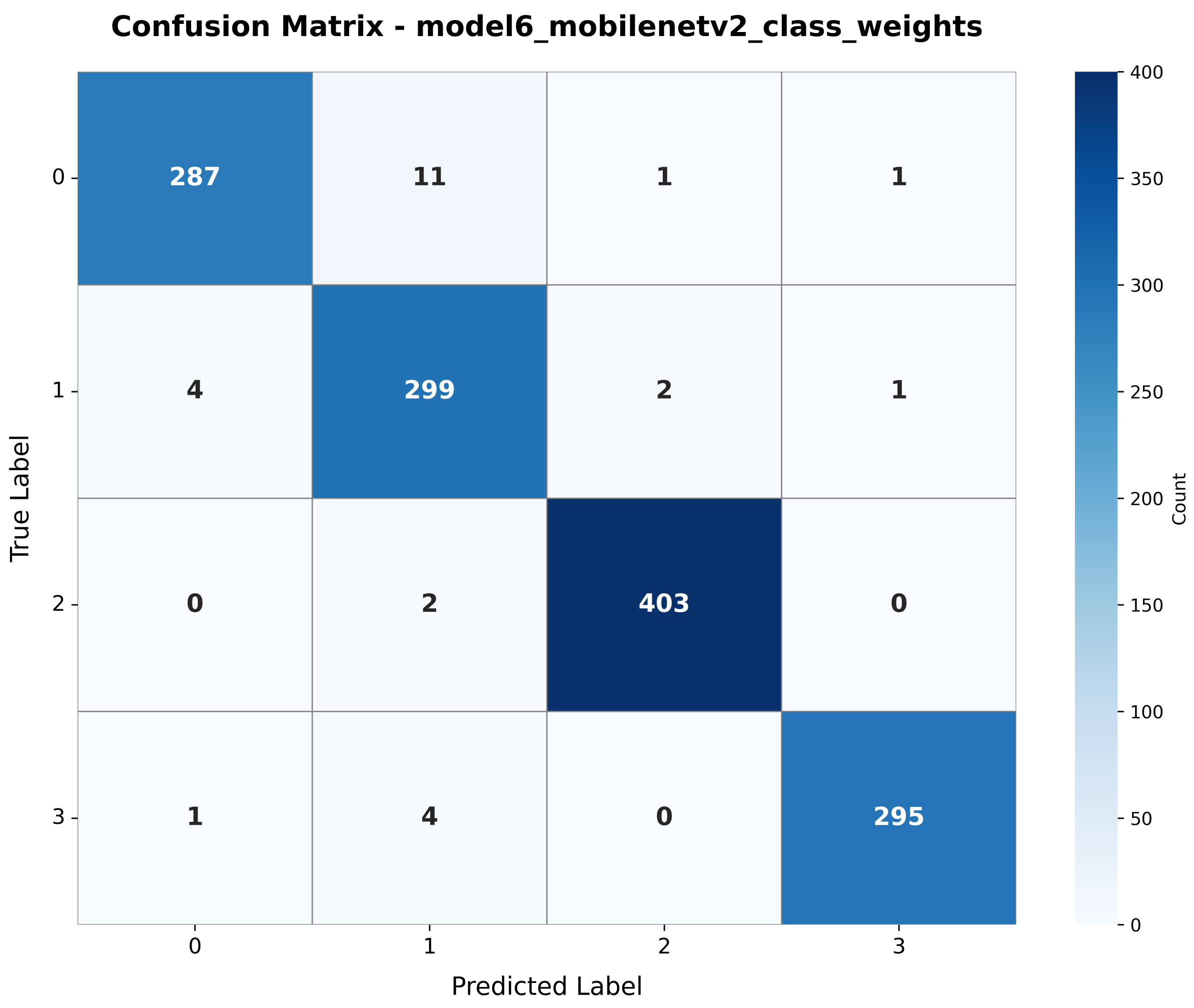

Figure S6: Confusion matrix of the MobileNetV2 model showing performance across Glioma, Meningioma, Pituitary, and No Tumor classes.

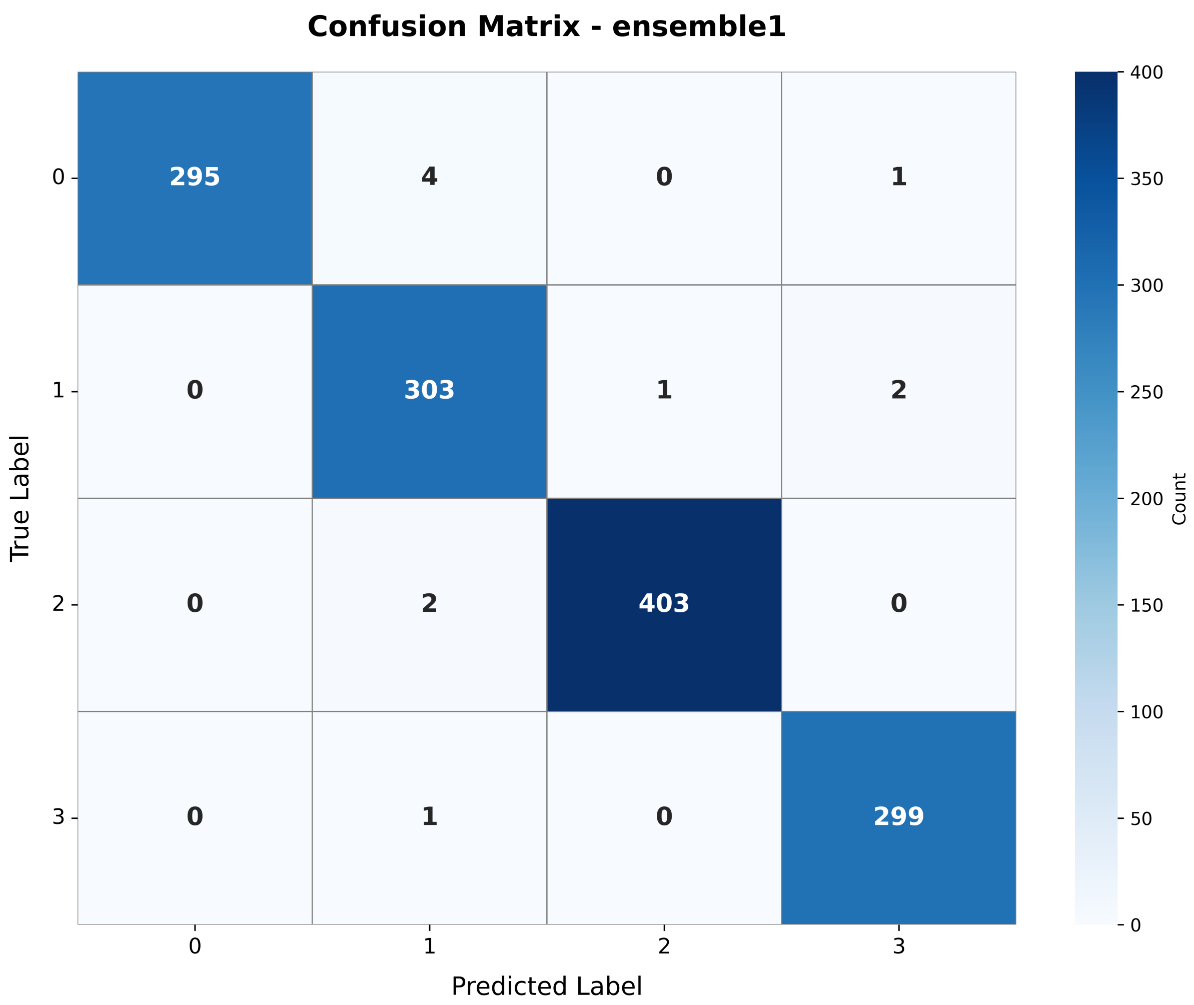

Figure S7: Confusion matrix of Ensemble Model 1 showing aggregated classification performance across all brain tumor classes.

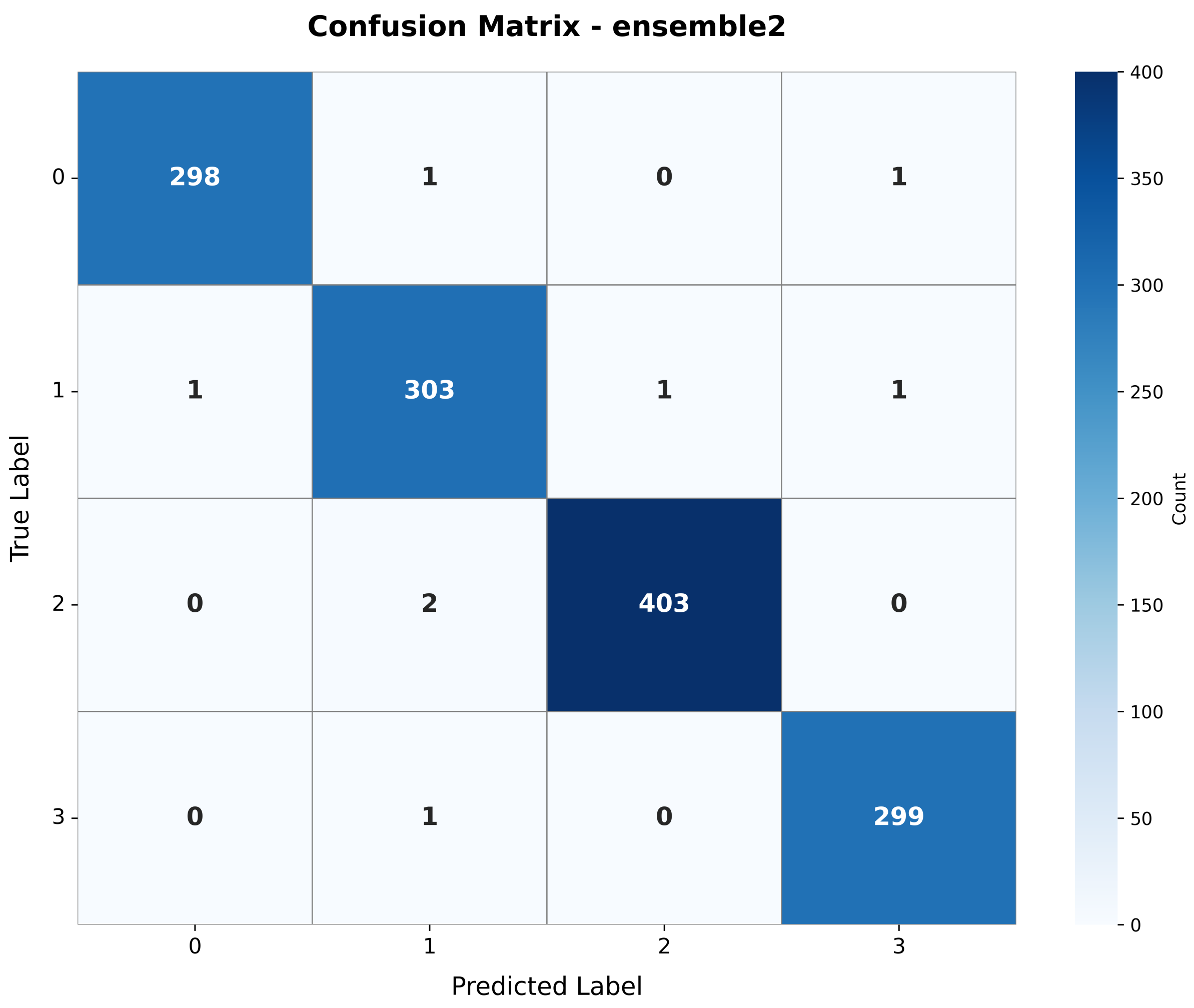

Figure S8: Confusion matrix of Ensemble Model 2 demonstrating improved classification stability through model fusion.

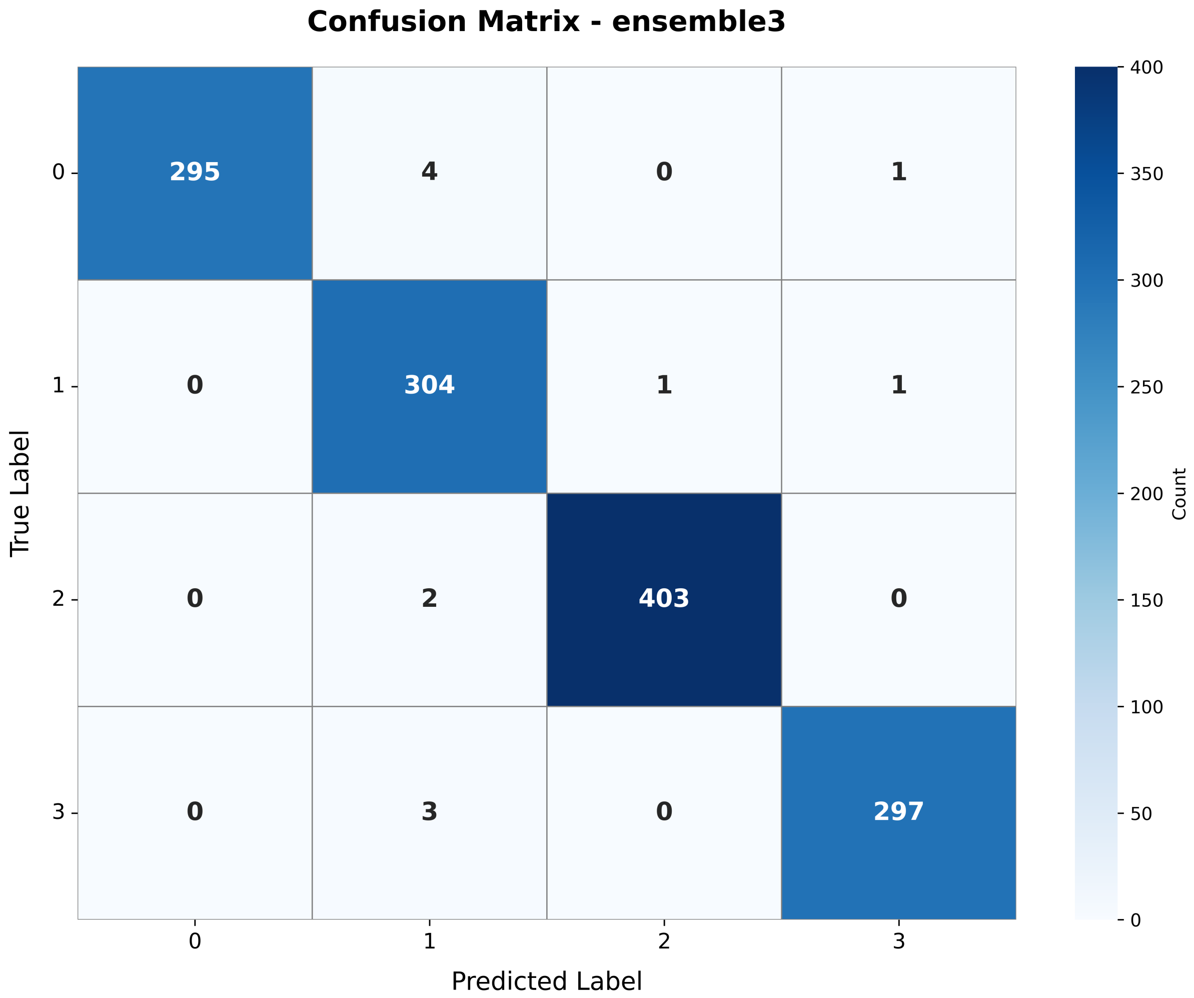

Figure S9: Confusion matrix of Ensemble Model 3 highlighting final fused predictions across all tumor categories.
